# An Ultrathin, Cyano‐Functionalized Copolymeric Memristor by iCVD Process for Driving Convolutional Neural Networks of High‐Resolution Images

**DOI:** 10.1002/advs.202511801

**Published:** 2025-11-27

**Authors:** Ji In Kim, Minsu So, Woo Jin Wang, Taehoon Kim, Eun Su Jeong, Kyumin Sim, Hamin Park, Sung Kyu Kim, Yong Goo Shin, Junhwan Choi, Min Ju Kim

**Affiliations:** ^1^ Department of Foundry Engineering Dankook University Yongin‐si 16890 Republic of Korea; ^2^ Department of Chemical Engineering Dankook University Yongin‐si 16890 Republic of Korea; ^3^ Department of Electronics and Information Engineering Division of Smart Energy Convergence Engineering Korea University Sejong 30019 Republic of Korea; ^4^ Department of Nanotechnology and Advanced Materials Engineering Sejong University Gwangjin‐gu 05006 Republic of Korea; ^5^ Department of Electronic Engineering Kwangwoon University Seoul 01897 Republic of Korea; ^6^ Department of Semiconductor Convergence Engineering Dankook University Yongin‐si 16890 Republic of Korea; ^7^ Department of Electronics and Electrical Engineering Dankook University Yongin‐si 16890 Republic of Korea

**Keywords:** compute in memory (CIM), convolutional neural network (CNN), deep learning, initiated chemical vapor deposition (iCVD)

## Abstract

For on‐chip learning, ideal weight storage elements should have scalability, data retention, symmetry and linear conductance modulation, and weight fine‐tuning capabilities. In this study, memristors are fabricated by employing cyano‐based ultrathin copolymer films (<10 nm) using 2‐cyanoethyl acrylate (CEA) and di(ethylene glycol) divinyl ether (DEGDVE) as functional monomers via an initiated chemical vapor deposition (iCVD) process, optimized to serve as a high‐performance device for convolutional neural networks (CNNs). The device achieves highly linear, symmetric, and multi‐level conductance modulation through precise control of polymer composition engineering. The switching characteristics and filament formation are controlled by varying the ratio of CEA and DEGDVE. In addition, the reliability and operation mechanism of the device are studied through non‐invasive observation of the conducting filament dynamics via electrical manipulation using ramp pulse series (RPS). Finally, image classification tasks ares performed on high‐resolution datasets such as Oxford 102 Flowers, Food‐101, and Stanford Cars by varying pulse amplitudes and durations to simulate conductance modulation such as potentiation and depression of weights in memristors. Utilizing various networks such as VGG‐X, ResNet‐X, and DenseNet, the proposed system demonstrated robust performance, achieving up to 88.39% classification accuracy, validating the efficiency of the memristor‐based CNN architecture in real‐world AI applications.

## Graphical abstract

Two‐terminal polymer memristors based on cyano‐functionalized copolymer films are developed via a solvent‐free, initiated chemical vapor deposition (iCVD) process for high‐resolution image classification. Molecularly engineered p(CEA‐co‐DEGDVE) films enable stable, linear, and symmetric conductance modulation, supporting multi‐level weight mapping in convolutional neural networks (CNNs). These results highlight the potential of iCVD‐based polymer memristors for energy‐efficient compute‐in‐memory (CIM) systems.

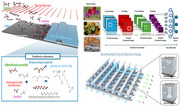

## 1 Introduction

**Figure 1:**
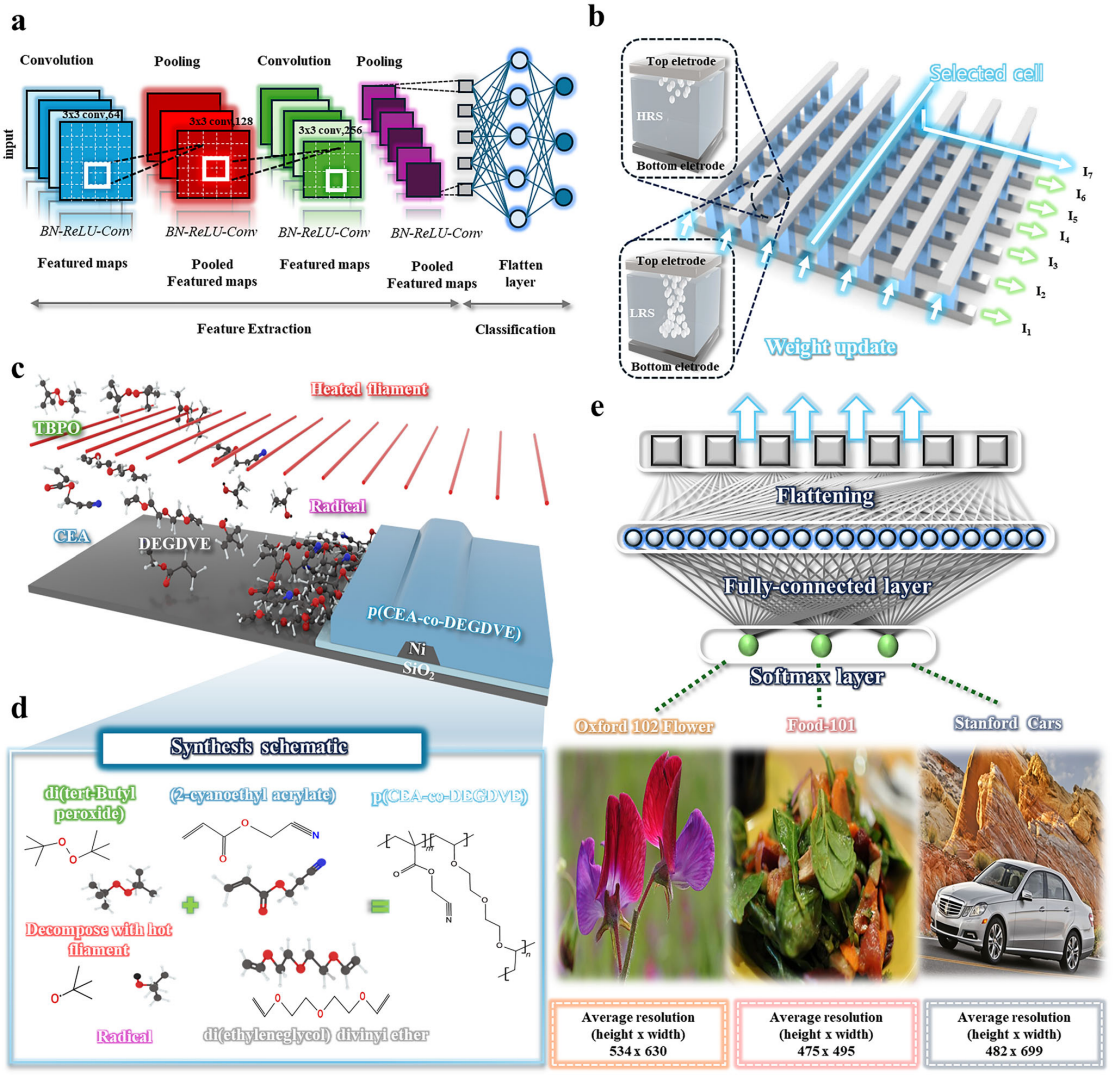
a) Schematic illustration of convolutional neural network (CNN) operations for high‐resolution image processing. b) Conceptual diagram of memristor crossbar arrays and device structure for in‐memory computation. c) Schematic of ultrathin copolymer film synthesis via initiated chemical vapor deposition (iCVD). d) Molecular structures of vaporized monomers used in iCVD. e) Schematic representation of weight mapping and learning process, where CNN‐extracted features are encoded into memristor conductance states through weight updates.

With the rapid advancements in artificial intelligence (AI) and machine learning (ML), convolutional neural networks (CNNs) have emerged as a core technology for processing high‐resolution images.^[^ ^1^, ^2^ ^]^ The demand for precise and efficient processing of large‐scale data continues to grow in diverse applications, including medical imaging and satellite image analysis.^[^ ^3^, ^4^ ^]^ In particular, CNNs have been widely adopted for large‐scale and high‐resolution image analysis, as convolution and pooling operations are highly effective for extracting key features (**Figure** **1**a). Increasing the depth of convolutional layers is generally regarded as an effective strategy to enhance CNN performance.^[^ ^5^, ^6^ ^]^ However, CNNs are constrained by the so‐called “memory wall,” a bottleneck caused by the physical separation of computation and memory in von Neumann architectures, which severely limits energy efficiency and scalability in conventional hardware implementations.^[^ ^7^, ^8^ ^]^ Among various candidate to overcome these challenges, two‐terminal memristors based on embedded non‐volatile memory (eNVM) are particularly attractive owing to their low power consumption, high scalability, and in‐memory computing capability (Figure 1b).^[^ ^9^ ^]^ When integrated into nanoscale crossbar arrays, memristors can efficiently encode multi‐level weight values as conductance states, thereby providing massive parallelism and enabling neural network acceleration for high‐resolution image processing tasks.^[^ ^10^, ^11^, ^12^, ^13^, ^14^ ^]^ To this end, both inorganic‐based materials and organic‐based materials have been actively investigated to realize multi‐level memristor systems. Inorganic memristors exhibit stable, fast, and robust resistive switching with high thermal stability, which ensures compatibility with CMOS processes.^[^ ^15^, ^16^ ^]^ Organic memristors provide mechanical flexibility, biocompatibility, and high integration density, making them suitable for flexible electronics and wearable systems.^[^ ^17^, ^18^, ^19^ ^]^ In particular, polymer‐based materials offer unique advantages owing to their versatile molecular design: functional groups can be precisely designed and incorporated to regulate metal–polymer interactions and conductive pathways.^[^ ^20^, ^21^, ^22^, ^23^ ^]^ However, conventional polymer memristors still suffer from device‐level non‐idealities, such as nonlinear and asymmetric switching behavior, limited conductance states, and poor cycle‐to‐cycle reproducibility, which hinder reliable in‐situ training and inference for high‐resolution image processing.^[^ ^24^, ^25^, ^26^ ^]^ Moreover, solution‐processed polymer films often face challenges, where dewetting, pinhole defects, and residual solvent impurities remain critical obstacles in securing uniform and scalable device fabrication.^[^ ^27^, ^28^ ^]^ In this study, we employed initiated chemical vapor deposition (iCVD) to design and fabricate copolymer‐based memristors optimized for memristor‐based CNN simulation. Specifically, we engineered a copolymer system comprising 2‐cyanoethyl acrylate (CEA), which introduces highly polar cyano groups to regulate metal–polymer interactions and facilitate controllable filament formation, together with diethylene glycol divinyl ether (DEGDVE), which acts as a crosslinking moiety to reinforce dielectric strength (Figure 1c,d).^[^ ^29^, ^30^ ^]^ The iCVD process offers a solvent‐free, vapor‐phase copolymerization strategy under precise stoichiometric control, enabling uniform and defect‐free thin films to be synthesized across the wafer scale. As a radical‐mediated polymerization process, it provides highly uniform copolymer layers with tunable chemical functionalities, thereby enabling the design of polymer switching films with improved electrical stability and reproducibility. A more detailed mechanism of the iCVD process is provided in Supplementary Information (Figure S1, Supporting Information).^[^ ^28^, ^31^ ^]^

Figure 1e schematically illustrates the weight mapping process in the memristor‐based CNN simulation framework for high‐resolution image classification. Input images are processed through convolution and pooling layers, with extracted features subsequently flattened into 1D vectors that are converted into digital weight values. These weights are then encoded into conductance states of memristor crossbar arrays via electrical signals, where each memristor corresponds to a specific weight magnitude.^[^ ^9^, ^32^, ^33^, ^34^ ^]^ As a result, software‐based CNN training outcomes can be simulated in hardware through spatial conductance distributions in the polymer films. By introducing polar functional groups at the molecular level and precisely controlling the composition with crosslinking moieties, we improved the device‐level switching behavior and achieved stable multi‐level weight modulation for CNN‐based image classification. Taken together, these findings suggest the strong potential of polymer‐based memristors as building blocks for high‐performance compute‐in‐memory (CIM) systems, contributing to the development of future AI accelerators and ultra‐low‐power edge computing applications.

## 2 Result and Discussion

### 2.1 Material Characterization of Copolymeric Thin Films

As shown in Table S1 (Supporting Information), we synthesized copolymers with three different compositions (CEA/DEGDVE = 4:1, 1:1, 1:8) and designated them as pC4D1, pC1D1, and pC1D8, respectively. Among the synthesized copolymers, pC4D1 exhibited the highest deposition rate (0.76 nm min^−1^). As the DEGDVE flow rate increased, the deposition rate gradually decreased, which can be attributed to the characteristic of non‐homo‐polymerizable DEGDVE.^[^ ^35^ ^]^ The electron‐donating property of the vinyl ether group increases the electron density, which shortens the lifetime of the formed radicals, thereby suppressing free radical polymerization. As a result, DEGDVE can only polymerize in the form of a copolymer by integrating into the CEA segments. Furthermore, in the iCVD process, polymerization occurs on the substrate surface where monomers adsorb. Thus, the adsorption amount of each monomer directly influences the surface composition of the copolymer. The vapor pressures of CEA and DEGDVE at 25 °C are 0.059 and 0.0059 Torr, respectively. This indicates that under the same substrate temperature, the adsorption concentration of CEA is significantly higher than that of DEGDVE. Consequently, the composition of the final iCVD copolymer does not directly correspond to the vapor flow ratio of the monomers, and the copolymer composition can only be tuned within a CEA‐rich regime.

**Figure 2:**
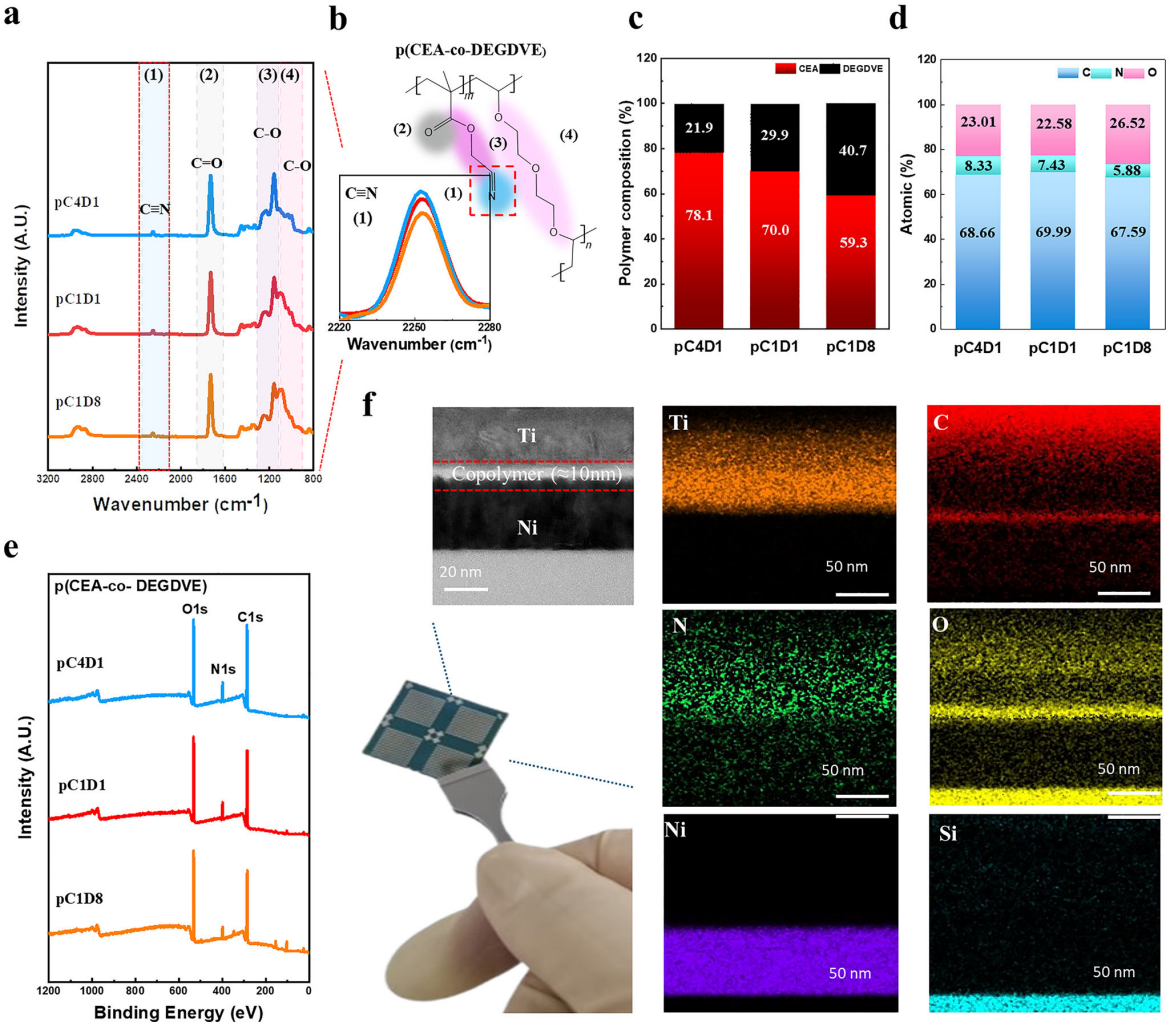
a) FTIR spectra of the copolymer films showing characteristic peaks of functional groups (1: C≡N, 2: C═O, 3: C─O in the ether group, 4: C─O in the ester group). The inset shows an enlarged view of the 2200–2300 cm^−1^ region. b) Chemical structure of the p(CEA‐co‐DEGDVE) copolymer. c) Monomer proportions of CEA and DEGDVE in each copolymer. d) Atomic percentages of the copolymer films determined from XPS analysis. e) XPS survey spectra of copolymer films. f) Cross‐sectional TEM image and EDS mapping of the p(CEA‐co‐DEGDVE) copolymer film.

**Figure** **2**a presents the chemical structure and FTIR spectra of the copolymers. In the spectra of all polymers, the C═C vinyl peak ≈1600 cm^−1^ was hardly detected, confirming the successful free radical polymerization via iCVD. In all polymers, characteristic peaks, such as a broad CH_2_ peak ≈2900–3000 cm^−1^, 1) the C≡N stretching vibration peak at 2250 cm^−1^, and 2) the C═O stretching vibration peak at 1730 cm^−1^, were observed, which are well consistent with the chemical structure of p(CEA‐co‐DEGDVE) copolymer.^[^ ^36^, ^37^ ^]^ In the case of C≡N, a change in the peak size was observed ≈2250 cm^−1^ in the spectra, indicating that the size of the C≡N peak increased due to the increase in cyanide as the CEA content increased (Figure 2b), which is fully consistent with previous observations. Similarly, as the DEGDVE flow rate increased, the intensity of the 3) 1160 cm^−1^ peak representing the ester group of CEA decreased, and the intensity of the 4) C─O─C ether peak (1090 cm^−1^) of DEGDVE gradually increased. All these observations indicate that the cyanide and ethylene glycol functional groups in the polymer thin film were not damaged during the polymerization process due to the mild conditions of the iCVD process.^[^ ^36^, ^37^ ^]^ To quantify the surface composition of the copolymer films, XPS analysis was conducted, and the atomic percentages are summarized in (Figure 2d). Figure S2 (Supporting Information) shows the high‐resolution XPS C1s and O1s spectra of the copolymers, where characteristic peak intensity changes indicate that the chemical composition of the films can be tuned according to the monomer flow ratio. Based on the XPS survey scan data (Figure 2e), the calculated CEA‐to‐DEGDVE ratios for each copolymer are shown in Figure 2c. Notably, the CEA fraction in the resulting copolymer films did not match the respective input flow ratios. This discrepancy may be attributed to the aforementioned poor homopolymerizability of the DEGDVE monomer. Collectively, the FTIR and XPS analyses consistently demonstrate that the chemical composition of the copolymer films can be effectively controlled by adjusting the monomer flow ratio in the iCVD process. Furthermore, cross‐sectional TEM imaging and EDS elemental mapping of the copolymer films (Figure 2f) revealed the formation of a uniform ultrathin (≈10 nm) layer between the Ni bottom and Ti top electrodes. The formation of a uniform ultrathin copolymer film and the stable elemental distribution throughout the thickness are further evidenced (Figures S3 and S4, Supporting Information). AFM analysis in Figure S5 (Supporting Information), which demonstrated sub‐nanometer surface roughness for all copolymer compositions, indicating smooth and uniform surface morphology consistent with the conformal coverage characteristics of the iCVD process. These features serve as a critical foundation for ensuring the stable and reliable operation of polymer‐based memristor devices.

### 2.2 Electrical Characterization and Optimization

**Figure 3:**
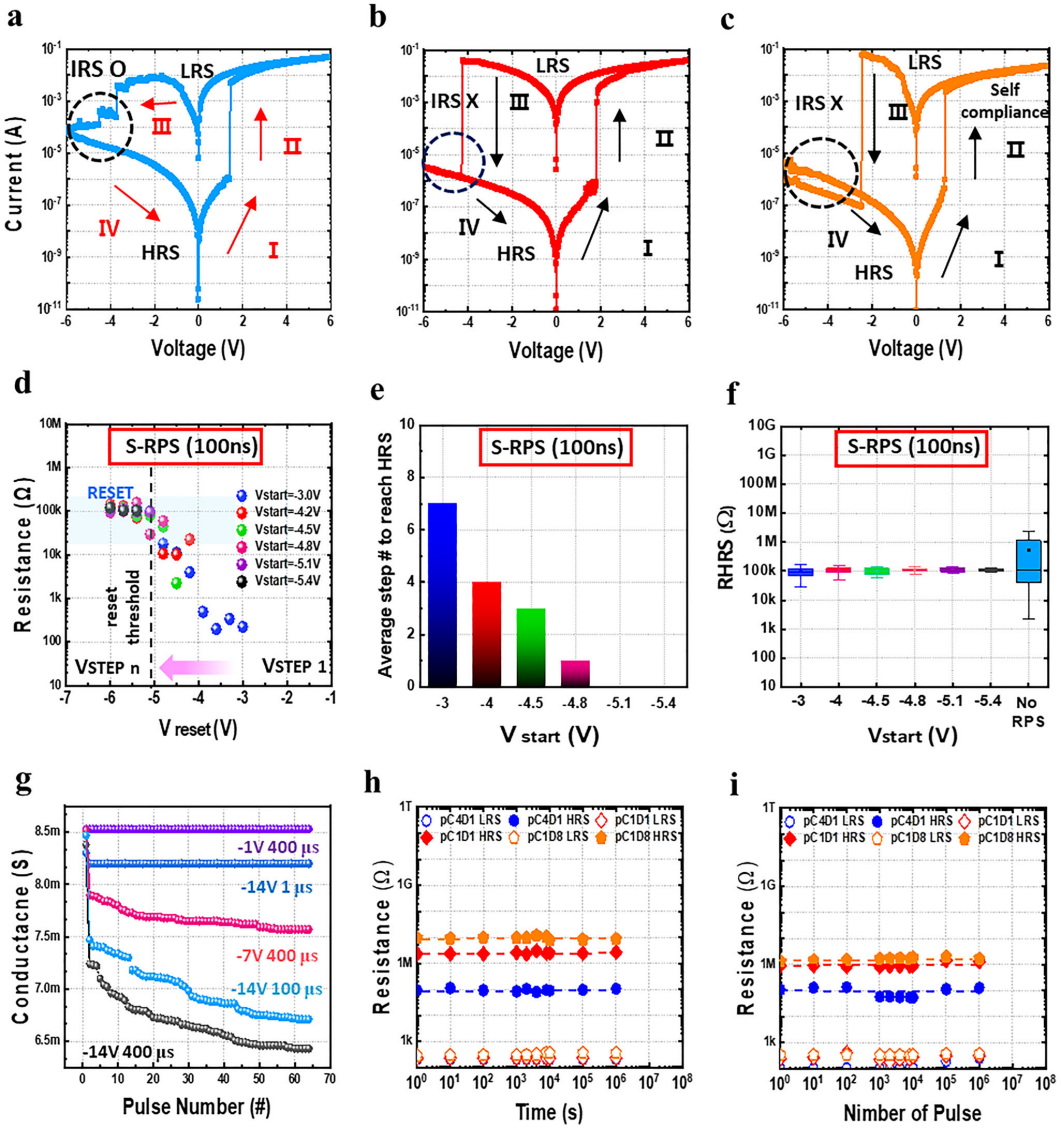
Electrical device operating characteristics a) pC4D1 b) pC1D1 c) pC1D8. d) RPS measurement of the switching resistance from LRS to HRS at every RPS step (pC4D1). e) Average number of steps required to reach HRS of pC4D1. f) Deviation of HRS after each Vstart of pC4D1 at the short term. g) Depression variation of the pC4D1 device with different pulse amplitudes and voltages. h) Retention performance at 85 °C. i) Endurance test results under −3 to −6 V pulses (100 ns).

The resistive switching behaviors of Ti/p(CEA‐co‐DEGDVE)/Ni‐based devices were characterized by pC4D1, pC1D1, and pC1D8. All electrical measurements were performed under an air atmosphere without any device encapsulation. The CF formation and bipolar switching behaviors were maintained generally consistent regardless of the composition ratio (**Figure** **3**a–c; Figure S6, Supporting Information). The curves presented in Figure 3a–c reflect the average behavior according to each composition ratio, and the most representative curves were selected. Figure S7a (Supporting Information) shows the average performance more precisely. The pC4D1 set voltage was 1.2 ± 0.5 V, pC1D1: 1.3 ± 0.5 V, and pC1D8: 1.2 ± 0.5 V, all values were within 2 V. On the other hand, the reset voltages for the three devices varied depending on their composition. Specifically, the pC4D1 device exhibited a reset voltage of −4.8 ± 0.4 V, while the pC1D1 and pC1D8 devices showed values of −3.7 ± 0.2 V and −2.8 ± 0.2 V, respectively. These results suggest that the reset voltage increases with increasing CEA content and that the conductive filament rupture becomes more difficult as the concentration of polar functional groups increases. As shown in Figure S7b (Supporting Information), as the CEA composition increases, the average HRS resistance decreased, and increased variability was observed. As further experiments, Figure S7c (Supporting Information) presents 7 × 7 color maps of HRS and LRS distributions measured at 0.1 V after 100 *I*–*V* cycles. This is interpreted as a large change in the HRS resistance due to the fact that the conducting filaments are not completely removed during the reset process, and unstable paths remain. The physical cause of the resistance variability in HRS is related to the filament gap size. In LRS, the filament is continuous and provides a conductive path between the electrodes. However, when it breaks during the reset process, a gap is created, and electrons must tunnel through the gap to reach the anode. The formation and rupture of filaments are governed by interactions between polymer functional groups and metal cations within the resistive switching layer. To clarify this mechanism, we schematically illustrate the resistive switching behavior based on two different cases depending on the concentrations of CEA and DEGDVE. To better understand the characteristics of the conducting filaments, the conduction mechanism as a function of temperature was investigated (Figure S8, Supporting Information). This analysis provides insight into the properties and physical processes of metal filaments under various thermal conditions. The detailed resistive switching mechanism, including the formation and rupture of conducting filaments depending on the relative concentrations of CEA and DEGDVE, is illustrated in Figure S9 (Supporting Information). This figure describes how compositional variations influence filament morphology, ion mobility, and switching behavior.

For pC4D1, unlike pC1D1 and pC1D8, an intermediate resistance state (IRS), which is a non‐uniform resistance change, occurs during the reset process when a DC bias is applied. As supported by the trap‐controlled SCLC behavior with a steep slope region in Figure S10 (Supporting Information), such non‐ideal conduction behavior tends to result in more asymmetric and nonlinear conductance updates during weight modulation, which is unfavorable for multi‐level conductance modulation. This means that as the filament becomes thicker, it is observed to be partially connected rather than completely separated during the reset process, and also similar tendency is also reflected in the bipolar switching characteristics of devices with aluminum as the top electrode (Figure S11, Supporting Information). This incomplete reset process narrows the CF gap, which ultimately reduces the ON/OFF ratio and causes variability in key switching parameters such as OFF‐state resistance and reset voltage. To explain this phenomenon, we verify the reliability and working mechanism of the reset voltage (Vreset) and OFF‐state resistance (HRS) through non‐invasive electrical manipulation using the ramp pulse series (RPS) method, which gradually increases the amplitude of the voltage pulse. Figure 3d shows the HRS resistance measured using a short‐term RPS (S‐RPS) of 100 ns. To induce a more uniform and stable OFF state, the Vstart value ranged from −3 to −6 V, and the resistance distribution was shown through Vstep, which increases the voltage range by −0.3 V. Figure 3e shows how the S‐RPS method is applied and shows the number of steps required to turn the device off from the starting voltage. When the on/off ratio is two digits or more, the device is considered to be off. Under S‐RPS conditions, −5.1 V was determined to be the optimal operating reset voltage as it was the lowest Vstart that could turn the device OFF without IRS (incomplete reset state). Figure 3f shows the resistance measured in the High‐Resistance State (R_HRS_) plot obtained by the RPS method. It can be seen that the dispersion is improved at all Vstarts compared to the device without the RPS application as a result of brief electrical stress applied to the dielectric. These results indicate that the uniformity of reset voltage and resistance value is improved, and the characteristics are stable compared to the existing method (No‐RPS) by using a short pulse (100 ns).

Figure 3g shows the conductance modulation at various negative voltages and amplitudes, highlighting the depression characteristics of the device. While the conductance is almost unchanged at −1 V (400 µs) and −14 V (1 µs), significant depression is observed when the voltage and pulse duration are varied to −7 V (400 µs) and −14 V (100 µs), indicating that filament modulation is initiated under stronger electric fields. The maximum conductance modulation is observed at the maximum applied voltage and pulse duration of −14 V (400 µs). This is in contrast to the conventional abrupt reset that occurs under a short −5.1 V pulse (100 ns), where the prolonged −7 V (100 µs) pulse enables a gradual conductance modulation. As shown in (Figure S12, Supporting Information), when large negative biases (below −7 V) are applied with long pulse durations (≥100 µs), localized Joule heating accelerates the drift of Ti ions inside the insulating matrix. These ions either recombine with residual metallic species or migrate toward the cathode, leading to the progressive rupture and partial reconstruction of the conductive filament (CF). Such irreversible structural modifications induce nonuniform resistance changes and explain the variable depression behavior observed in the RPS operation. Therefore, based on this variable dropout behavior, the maximum conductance modulation occurring at −14 V (400 µs) provides a much wider and more stable dynamic range, especially under high‐voltage operation (−14 V/14 V), as shown in Figure S13 (Supporting Information), and can express a greater number of weighting states.

For application to artificial neural networks, memristors should exhibit both excellent analog switching and stable multi‐level retention characteristics to ensure reliable conductance states. The p(CEA‐co‐DEGDVE) based memristor maintained retention over 10^6^ s at 85 °C without significant degradation (Figure 3h). and showed stable LRS/HRS even after repeated set/reset operations with 100 ns pulses (Figure 3i). In addition, Figure S14 (Supporting Information) presents the cumulative distribution function (CDF) analyses of both cycle‐to‐cycle (C‐to‐C) and device‐to‐device (D‐to‐D) resistance variability for all compositions, confirming narrow resistance distributions in both HRS and LRS. The corresponding statistical parameters obtained from these analyses are summarized in Tables S2 and S3 (Supporting Information). These behaviors enable reliable distinction of multiple conductance states for neural learning. Overall, these results confirm that p(CEA‐co‐DEGDVE) memristors combine stability and analog tunability, making them promising candidates for implementing artificial neural networks.

### 2.3 Device Level: Conductance Modulation Characteristics

**Figure 4:**
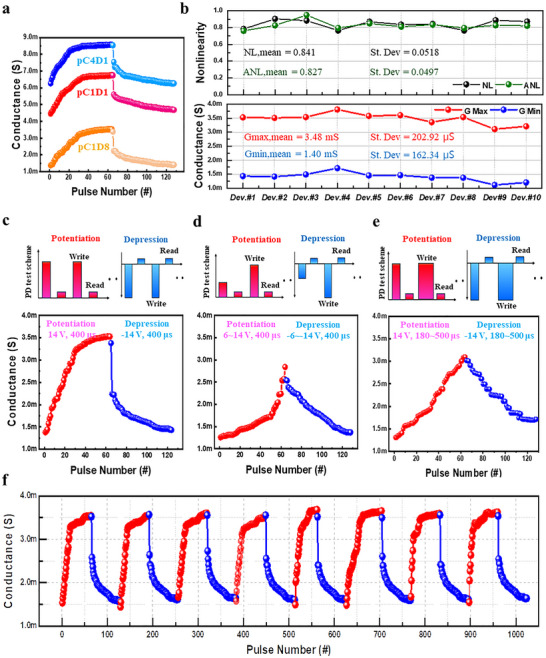
a) Comparison of potentiation and depression (P/D) curves according to copolymer composition. b) Nonlinearity (NL), asymmetry (ANL), and conductance range of pC1D8 devices. c–e) Conductance modulation behavior of pC1D8 memristors under multiple pulse conditions: c) Identical pulses, d) Non‐Identical Pulse I (voltage amplitude variation), and e) Non‐Identical Pulse II (pulse width variation). f) Endurance performance over repeated 64‐step potentiation/depression cycles.

To ensure applicability in artificial neural network systems, it is essential to demonstrate both analog conductance modulation and reliable endurance characteristics.^[^ ^38^ ^]^ In this section, we fit experimental weight update data and extract device parameters for use in the simulator. As shown in Figure S15 (Supporting Information), the device exhibited clear long‐term potentiation (LTP) behavior under repeated pulse trains, showing a gradual and sustained conductance increase without rapid decay after stimulation. This confirms the device's suitability for stable long‐term conductance potentiation and depression in artificial neural network (ANN) applications. **Figure** **4**a shows the excellent analog conductivity modulation characteristics of all copolymer memristors. All were measured by applying 64 pulses of 14 V (potentiation) and −14 V (depression) with the same pulse width of 400 µs. All devices demonstrated stable and reproducible performance, supporting their capability of multi‐level weight update for memory computing.

For high‐performance artificial neural network (ANN) applications, it is essential to support analog conductance updates and endurance while minimizing nonlinearity and asymmetry in conductance modulation. The non‐idealities are typically caused by abrupt formation or rupture of conductive filaments, resulting in sudden conductance modulation that degrades learning accuracy. Based on the previous section discussing reset voltage uniformity, operating voltage, and off‐state resistance stability, the pC1D8‐based memristor was selected as the optimal composition for evaluating conductance tunability characteristics. The weight modulation performance of pC1D8 devices was evaluated by analyzing the nonlinearity (NL), asymmetric nonlinearity (ANL), and conductance range (Gmax,Gmin) across multiple devices (Figure 4b). The nonlinearity of the weight update can be defined quantitatively as:^[^ ^39^ ^]^

(1) $$NL=\frac{\max\left|G_{P}\left(n\right)-G_{D}\;\left(65-n\right)\right|}{Gmax-Gmin}\;for\;n=1\;to\;64$$

where G_P_(n) and G_D_(n) are the conductance values after the nth potentiation pulse and nth depression pulse, respectively. The Gmax and Gmin represent the maximum conductance value after 64 potentiation pulses and the minimum conductance value at the initial state, respectively.

The asymmetry nonlinearity factor (ANL) of the device can be quantitatively defined as:^[^ ^40^ ^]^

(2) $$ANL=\frac{G_{P}\left(\frac{N}{2}\right)-G_{D}\left(\frac{N}{2}\right)}{\left(G_{max}-G_{min}\right)}$$

where G_max_, G_min_, G_P_(N/2), and G_D_(N/2) represent the maximum conductance, conductance, minimum, median value of potentiation, and median value of depression, respectively.

The extracted data showed very uniform and consistent linearity with an average NL of 0.841 (±0.0518) and an ANL of 0.827 (±0.0497). In addition, the average maximum and minimum conductance values ​​were 3.48 mS (±202.92 µS) and 1.40 mS (±162.34 µS), Furthermore, Figure S16 (Supporting Information) presents a comparison of NL and ANL across different compositions, followed by additional experiments designed to further improve the performance of the most promising composition. Respectively, demonstrating a stable conductance range. These results demonstrate that pC1D8 based on the p(CEA‐co‐DEGDVE) configuration exhibits reproducible and stable in‐memory characteristics with a uniform conductance range in artificial neural network (ANN) computing systems. In this sense, NL, ANL, and the available conductance range (Gmax–Gmin) act as essential enablers that set the feasible performance envelope for learning, i.e., they determine how linear, symmetric, and expressive weight updates can be under a given pulse scheme (Figure 4c–e; Tables S2–S4, Supporting Information). At the same time, whether a system actually reached the upper part of this envelope depends on array‐level variability and operation (C‐to‐C / D‐to‐D spreads), as well as on architecture‐ and dataset‐specific sensitivities that shape error propagation during training.

In addition, to enable linear and symmetric conductance behavior, it is crucial to control filament dynamics by optimizing external pulse parameters such as amplitude, width, and intervals without modifying the RS layer or device structure. In variable pulse schemes, optimizing these pulse parameters enables the linearity and symmetry of in‐memory weight updates to be effectively enhanced, which is critical for accurate learning. To validate the effect of pulse parameter optimization, we tested the effect under three different pulse schemes as shown in Figure 4c–e. Detailed illustrations of each pulse configuration are provided in Figure S17 and Table S4 (Supporting Information), including variations in amplitude, width, and timing intervals for potentiation and depression sequences. In the identical pulse scheme (Figure 4c), potentiation and depression voltage amplitudes were fixed at +14 and −14 V with a 400 µs pulse interval. Conductance increased with pulse numbers during potentiation and decreased during depression. The calculated NL and ANL values ​​were up to 0.78 and 0.76, respectively, indicating linearity and symmetry under the same pulse conditions. In the Non‐Identical Pulse‐I scheme (Figure 4d), variable voltage amplitudes ranging from 6 to 14 V in 0.125 V steps were applied with a constant pulse width of 400 µs. The calculated NL and ANL values ​​are up to 0.38 and 0.19, respectively, showing improved linearity and symmetry compared to the same pulse scheme. Finally, the Non‐Identical Pulse‐II scheme (Figure 4e) employed a fixed voltage amplitude of 14 V while increasing the pulse width from 180 to 500 µs in 5 µs steps. This configuration further reduced the NL and ANL values to 0.19 and 0.04, approaching the ideal NL and ANL values of zero and representing one of the smallest among all reported two‐terminal and three‐terminal memristors (Table S5, Supporting Information). These results confirm that optimizing pulse parameters such as voltage amplitude and width enables more linear and symmetric conductance modulation. Notably, the *I*–*V* characteristics remained unchanged even under high‐voltage conditions and extended pulse durations (Figure S18, Supporting Information).

Moreover, as shown in Figure 4f, after applying more than 1000 consecutive pulses consisting of repeated 64‐step potentiation and depression sequences, the pC1D8 device maintained excellent endurance with minimal degradation in conductance modulation. This robustness is attributed to its optimized copolymer composition and high cross‐linking density, which contribute to stable filament dynamics under repetitive switching cycles.

### 2.4 CNN‐Based High‐Quality Image Recognition Simulation Accuracy

**Figure 5:**
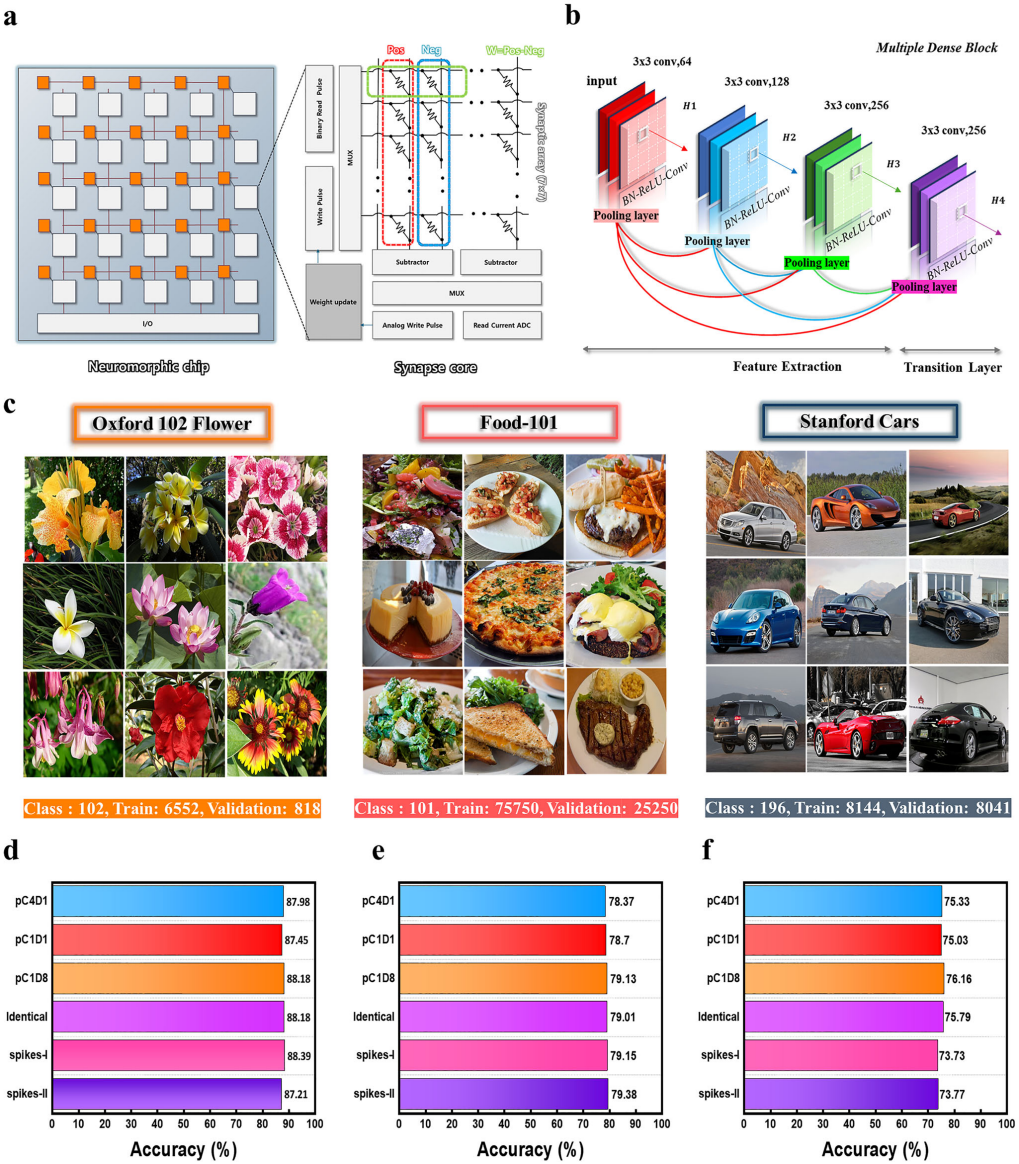
a) Equivalent hardware layout for neural network emulation, showing peripheral circuitry and crossbar‐based memristor weight mapping for on‐chip CNN training. b) The architecture of DenseNet is used for efficient feature extraction. c) Representative high‐resolution datasets used in this study (Oxford 102 Flowers, Food‐101, and Stanford Cars). Classification accuracy obtained using DenseNet‐121 as the baseline model for d) Oxford 102 Flowers, e) Food‐101. f) Stanford Cars.

In artificial neural network (ANN) systems, the performance of memristive weight arrays is typically evaluated through system‐level simulation. To illustrate the relationship between device‐level weight modulation and convolutional neural network (CNN) architecture, **Figure** **5**a presents an equivalent circuit diagram for implementing an analog ANN simulation system. In this scheme, two memristors are configured as one artificial weight storage unit to encode both positive and negative weights. The differential weight value is obtained by subtracting the output current of the negative branch from that of the positive branch, and the resulting signal is digitized through an analog‐to‐digital converter (ADC). Weight updates are applied using analog write pulses, whereas binarized read pulses are employed to transmit binary signals. In particular, DenseNet, a high‐performance CNN architecture, was employed to evaluate the system‐level impact of our memristive devices within the memristor‐based CNN simulation framework (Figure 5b). By leveraging dense residual connections, DenseNet achieves high accuracy while maintaining computational efficiency, enabling effective training of deep CNN models on complex, high‐resolution image datasets. To validate the performance of the proposed device in deep learning applications, it is crucial to demonstrate both stable operation across various neural network architectures and high classification accuracy on diverse image datasets. As an initial step, we conducted image classification experiments on the MNIST and CIFAR‐10 datasets using shallow convolutional neural networks adopted from previous studies (Figure S19a,c, Supporting Information). These experiments focused on evaluating the impact of different composition ratios and spike shapes. As shown in Figure S19b,d (Supporting Information), the proposed device outperforms conventional counterparts on both datasets while maintaining consistent operational stability. These results suggest that the proposed device can achieve enhanced even under identical network architectures.

However, the models employed in prior studies^[^ ^41^, ^42^, ^43^, ^44^ ^]^ are relatively simple and typically consist of shallow CNNs with limited depth, and do not adequately reflect the complexity of neural networks commonly used in real‐world image classification tasks. Therefore, the results shown in Figure S19b,d (Supporting Information) alone are insufficient to comprehensively assess the device performance across a wide range of modern deep learning architectures. To validate the performance of the proposed device in practical image classification tasks, we selected three public datasets with distinct characteristics: Oxford 102 Flowers (average resolution 534 × 630), Food‐101 (475 × 495), and Stanford Cars (482 × 699).^[^ ^45^, ^46^, ^47^ ^]^ These datasets encompass diverse visual properties and complexity, providing a robust basis for evaluating how consistently the proposed model performs across different image classification tasks (Figure 5c). Additional evaluations using multiple neural network architectures were conducted with the Oxford 102 Flower dataset, and the detailed results are provided in the Supplementary Information (Figure S20, Supporting Information). As shown in Figure S20 (Supporting Information), DenseNet‐121 achieved the highest accuracy among the tested architectures and was therefore selected as the baseline model for subsequent experiments on Oxford 102 Flowers, Food‐101, and Stanford Cars. As illustrated in Figure 5d–f the proposed device consistently delivered high performance across all datasets. Note, however, that accuracy rankings across different neural architectures do not always preserve the ranking implied by device‐level metrics. This is expected under a multi‐scale view: device metrics define a favorable update envelope, while *i*) array‐level variability (C‐to‐C/D‐to‐D), *ii*) pulse‐scheme dependence, and *iii*) architecture–dataset sensitivity jointly determine where each system operates within that envelope. Hence, device metrics provide strong directional guidance but are not, by themselves, rank‐preserving predictors across heterogeneous models and tasks. (Figure 5d–f; Tables S2–S4, and S6, Supporting Information).

## 3 Conclusion

To support CNN applications, we fabricated a two‐terminal memristor in the form of a crossbar array using an ultrathin defect‐free copolymer film p(CEA‐co‐DEGDVE) manufactured by an iCVD process, thereby realizing an ideal multi‐level conductance cell suitable for memristor‐based CNN simulation and in‐memory computing. The difference in conductivity properties depending on the chemical composition of the copolymer dielectric is due to the variation in the gap size of the conducting filaments, which is controlled by the incorporation of highly polar cyano‐functionalized CEA, facilitating strong interaction with metal ions. In addition, we optimized linearly and symmetrically weighted update characteristics of the device by varying voltage and pulse parameters. As a result, a high‐quality CNN simulation based on potentiation and depression characteristics using the DenseNet‐121 architecture achieved a classification accuracy of up to 88.39%. This study not only proposes suitable switching matrix materials for realizing a copolymer‐based in‐memory array but also paves the way for fabricating high‐density, high‐performance in‐memory devices. Additionally, it suggests effective neural architecture design, learning mechanism development and hardware optimization. These results demonstrate the high potential of copolymer‐based memristor devices for future hardware implementations of artificial neural networks (ANNs). Importantly, our study clarifies that device‐level metrics (NL, ANL, Gmax–Gmin) are indispensable for enabling reliable and expressive multi‐level weight updates and thereby constraining the attainable accuracy range, whereas the realized accuracy reflects additional shaping by array variability, pulse programming, and architecture–dataset factors.

## 4 Experimental Section

### Polymer Film Deposition and Characterization

To produce ultra‐thin CEA and DEGDVE composite films of 10 nanometers or less, CEA (>95.0%) was purchased from Tokyo Chemical Industry. DEGDVE (99%) and TBPO (98%) were purchased from Sigma–Aldrich, and these materials were purified without additional purification. The material was heated to generate a steady vapor stream and then injected into an iCVD chamber system (ISAC Research Co, Ltd). The CEA and DEGDVE were heated at different source temperatures for each composition. Specifically, the source temperatures were set to 70 and 80 °C for pC4D1, 70 and 50 °C for pC1D1, and 70 and 80 °C for pC1D8, respectively. During this process, the chamber pressure and substrate temperature were maintained at 10 mTorr and 30 °C to maintain the same process conditions. Additionally, the filament temperature was kept the same at 200 °C to ensure decomposition of the initiator (TBPO) and induce radical formation.

The functionality of the dielectric was assessed using Fourier Transform Infrared (FTIR) spectroscopy (Alpha, Bruker Optics, USA) in absorbance mode, revealing the generalized molecular structure and probe microscopy to analyze the deposited polymer thickness and synthesized polymer surface. 5 µm × 5 µm AFM images were taken using (XE‐100, Park Systems, Korea). Additionally, chemical bonds and atomic concentrations by composition were analyzed using X‐ray Photoelectron Spectroscopy (XPS) (NEXSA, ThermoFisher Scientific, USA). Before XPS analysis, the adsorbed particles were removed from the surface of the polymer film using N_2_ gas.

### Device Fabrication and Electrical Characterization

The p(CEA‐co‐DEGDVE) based memristor was fabricated with a crossbar architecture on a 500 nm thick SiO_2_ substrate, and the Ni (BE) bottom electrode was thermally deposited under the p(CEA‐co‐DEGDVE) film. Monolayer p(CEA‐co‐DEGDVE) (≈10 nm) was sequentially synthesized and deposited on the electrode. The top electrode of Ti (TE) was finally thermally deposited on the substrate. All electrodes were patterned with a shadow mask with an area of 200 × 200 µm^2^. For visualization of copolymer‐based devices, cross‐sectional images and atomic profiles were obtained by HAADF‐STEM measurements using EDS mode.

The electrical properties were then evaluated after one week of exposure to an air environment. Electrical characterization was performed using a B1500A semiconductor device analyzer (Keysight Technologies, USA) within (MSTECH, Korea). The values of the low resistance state (LRS) and high resistance state (HRS) are based on the current measured at 0.1 V. DC sweep and pulse measurements were performed using Keysight‐B1525A SP GU (pulse generator).

### Statistical Analysis

Electrical data were obtained from both individual device measurements and device arrays. For each composition, at least five independent device batches were evaluated. In single‐cell measurements, each device was subjected to 100 consecutive switching cycles to evaluate cycle‐to‐cycle (C‐to‐C) variability. In array‐level tests, a 7 × 7 crossbar array (49 cells) was measured to assess device‐to‐device (D‐to‐D) variability. Data are presented as the coefficient of variation (CV) is defined as the ratio of the standard deviation (σ) to the mean value (µ) for both HRS and LRS, and the worst case in switching window margin $\left(M_{i\_worst}\right)$ is calculated as 10*log*_10_( *w_i_* =  1), where the worst case in the switching window is *w*_*i*  =  1_ = *R*_*HRS*,*i* = 1_ /*R*_*LRS*,*i*  =  1_. Representative curves are shown together with averaged results across the tested devices.

### Datasets

The approach was validated using five benchmark datasets: MNIST, CIFAR‐10, Oxford 102 Flowers, Stanford Cars, and Food‐101. The MNIST dataset consists of 60000 training and 10000 testing grayscale images of handwritten digits, each sized 28 × 28 pixels, collected from 250 individuals. CIFAR‐10 contains 50000 training and 10000 test images of 32 × 32 pixels, spanning ten object categories. Oxford 102 Flowers provides 8189 images across 102 flower species, which was randomly divided into training and test sets with an 8:1 ratio. Stanford Cars includes 16185 images from 196 car categories, evenly split into 8144 training and 8041 test samples. Finally, Food‐101 comprises 101000 images from 101 food categories, with 750 training and 250 test images per class.

## Conflict of Interest

The authors declare no conflict of interest.

## Author Contributions

M.J.K. and J.C. designed and supervised the experiments. Y.G.S. developed a CNN‐based image recognition model using advanced networks and improved its accuracy. S.K.K. precisely analyzed the sample surface and microstructure and provided critical insights. H.P. supported the additional experimental measurements and validated the outcomes. J.I.K. performed the majority of the experiments, analyzed the data, and wrote the paper. M.S. established the chemical analysis and model and wrote the paper. W.J.W. performed the experiments using high‐quality image recognition simulations. T.K. performed additional measurements and edited the manuscript. E.S.J. performed some of the experiments. K.S. assisted with experimental measurements. All authors read and approved the final manuscript. J.I.K., M.S., W.J.W., and T.K. equally contributed to this work.

## Supporting information



Supporting Information

## Data Availability

The data that support the findings of this study are available from the corresponding author upon reasonable request.
