# Supplementary material for: An Ultrathin, Cyano‐Functionalized Copolymeric Memristor by iCVD Process for Driving Convolutional Neural Networks of High‐Resolution Images
Source: Adv Sci (Weinh). 2025 Nov 27;13(8):e11801. doi: 10.1002/advs.202511801 (PMC12884729; doi:10.1002/advs.202511801)
Supplement: Supplementary file 1 — Supporting Information [file ADVS-13-e11801-s001.docx]

Supporting Information

An Ultrathin, Cyano-Functionalized Copolymeric Memristor by iCVD Process for Driving Convolutional Neural Networks of High-Resolution Images

Ji In Kim^1$^, Minsu So^1$^, Woo Jin Wang^3$^, Taehoon Kim^1$^, Eun Su Jeong^2^, Kyumin Sim^5^ Hamin Park^5^, Sung Kyu Kim^4^, Yong Goo Shin^3^*, Junhwan Choi^1,2^*, Min Ju Kim^1,6,7^*

^1^ Department of Foundry Engineering, ^2^Department of Chemical Engineering, Dankook University

^3^Department of Electronics and Information Engineering, Korea University

^4^Department of Nanotechnology and Advanced Materials Engineering, Sejong University

^5^Department of Electronic Engineering, Kwangwoon University

^6^Department of Semiconductor Convergence Engineering, ^7^Department of Electronics & Electrical Engineering, Dankook University

^$^First author: Ji In Kim, Minsu So, Woo Jin Wang, Taehoon Kim

^*^Corresponding author: Min Ju Kim, E-mail: [minju9062@dankook.ac.kr](mailto:minju9062@dankook.ac.kr)

Junhwan Choi, E-mail: jhchoi2301@dankook.ac.kr

Yong Goo Shin, E-mail: ygshin92@korea.ac.kr


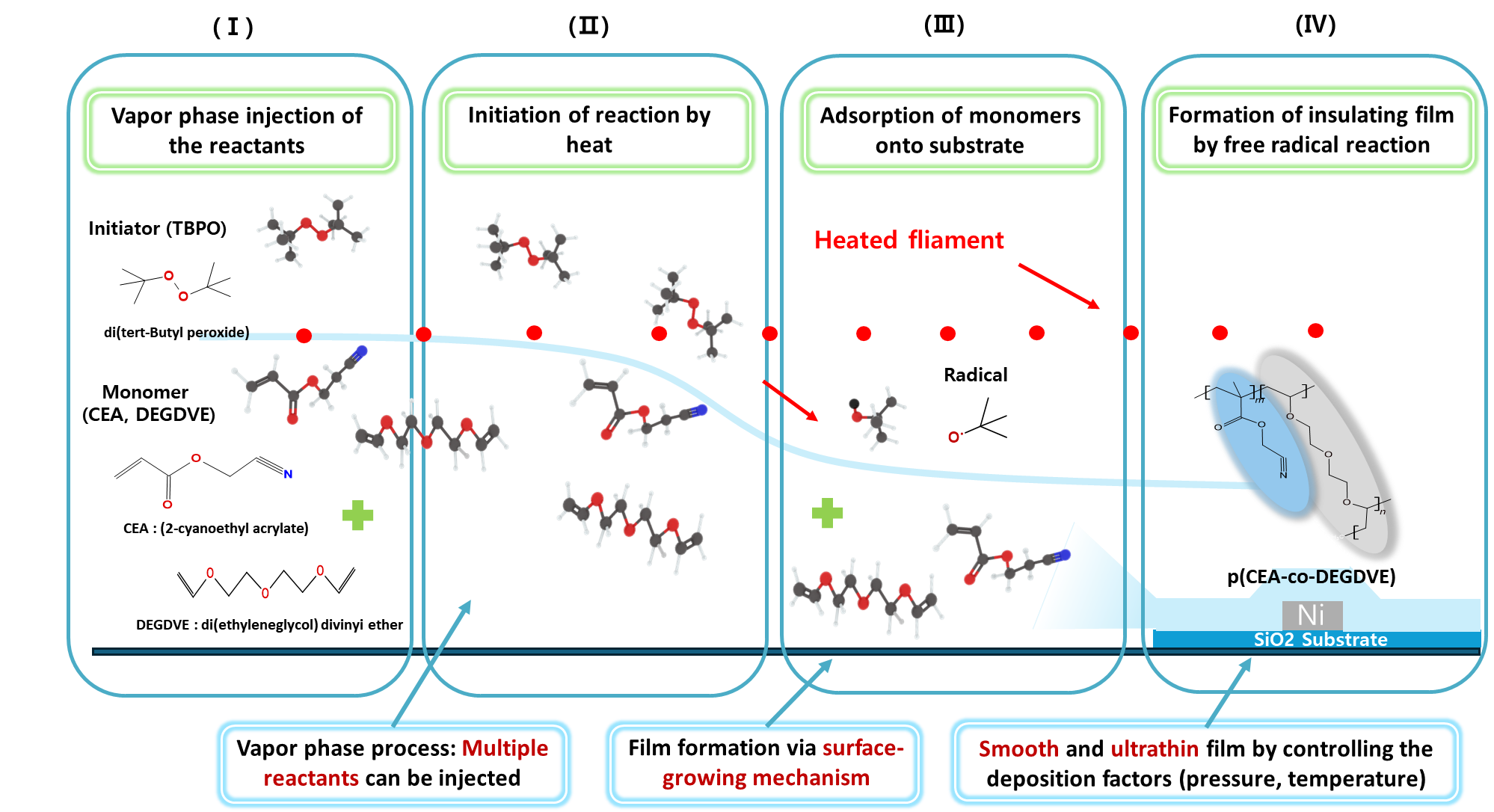


Figure S1. p(CEA-co-DEGDVE) process mechanism via iCVD process.

(Ⅰ) A volatile monomer(CEA,DEGDVE) and an initiator(TBPO) are simultaneously injected into the chamber as a gas phase, (Ⅱ) The initiator is thermally decomposed by a high-temperature filament (200°C) to form radicals, and the monomer is adsorbed onto a substrate at a low temperature (30°C). (Ⅲ) The highly reactive radical species move to the monomer adsorbed onto the substrate and transfer the radical to the Vinyl group (CH_2=CH-) in the monomer, (Ⅳ) Polymerization reaction and thin film formation occur simultaneously.

Table S1. process parameter of CEA-DEGDVE synthesis, including monomer flow rate, deposition rate and atomic percentages of the resulting polymer films.

| Name | CEA/DEGDVE Flow Rate Ratio (sccm) | | | CEA/DEGDVE flow rate ratio | Deposition Rate (nm/min) | CEA/DEGDVE (in polymer film) |
| --- | --- | --- | --- | --- | --- | --- |
|  | CEA | DEGDVE | TBPO |  |  |  |
| pC4D1 | 0.78 | 0.21 | 0.74 | 3.6:1 | 0.76 | 78.1:21.9 |
| pC1D1 | 0.64 | 0.46 | 1.73 | 1.38:1 | 0.57 | 70.0:29.9 |
| pC1D8 | 0.21 | 1.73 | 0.78 | 1:8 | 0.25 | 59.3:40.7 |


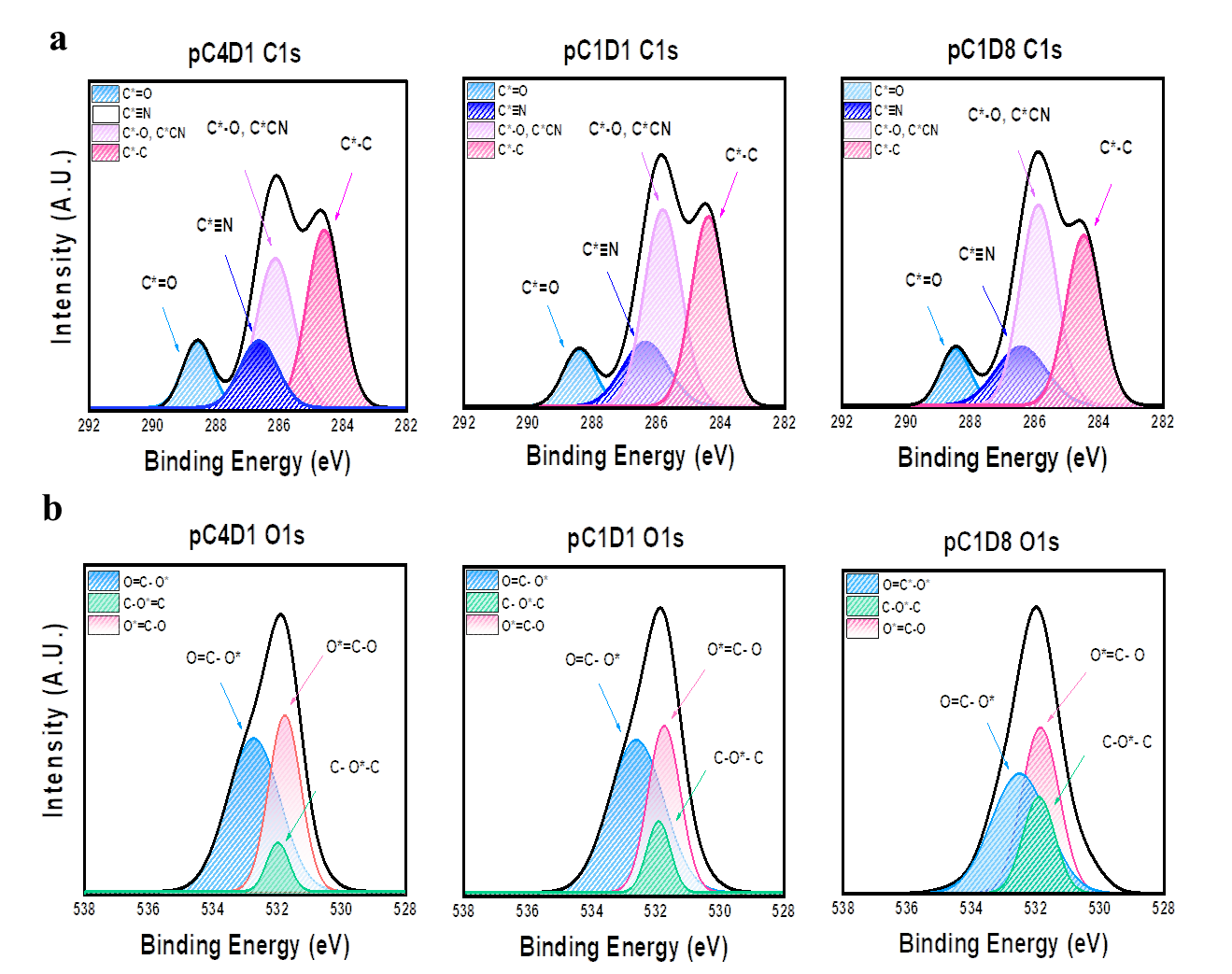


Figure S2. XPS survey spectra (a) High-resolution C1s spectra. (b) High-resolution O1s spectra.

Figure S2 shows the XPS C1s and O1s spectra of the copolymers. The carbon bond peaks in the high-resolution spectrum are as follows: 288.5(C*=O) eV, 286.7 (C*≡N) eV, 286.1(C*−O, C* CN) eV, and 284.8(C*−C) eV. C*≡N peaks, which also matched well with the FTIR analysis results. The C*=O and C*≡N peaks are detected due to the CEA monomer flow rate, and the area of the C*=O peak was the same as that of the C*≡N peak in all spectra (1). Additionally, because a larger amount of C*−O exists in DEGDVE, the intensity of the overlapped peak of C*−O and C*−CN increases as the DEGDVE flow rate increases. The chemical bonding of oxygen is as follows: 532.7(O=C−O*) eV, 532(O*=C−O) eV, 531.8(C−O*−C) eV. Similarly, the peaks of O=C−O* and O*=C−O have the same area in all O1s spectra. It was confirmed that the intensity and area of the C−O*−C peak increased as DEGDVE flow rate increased.


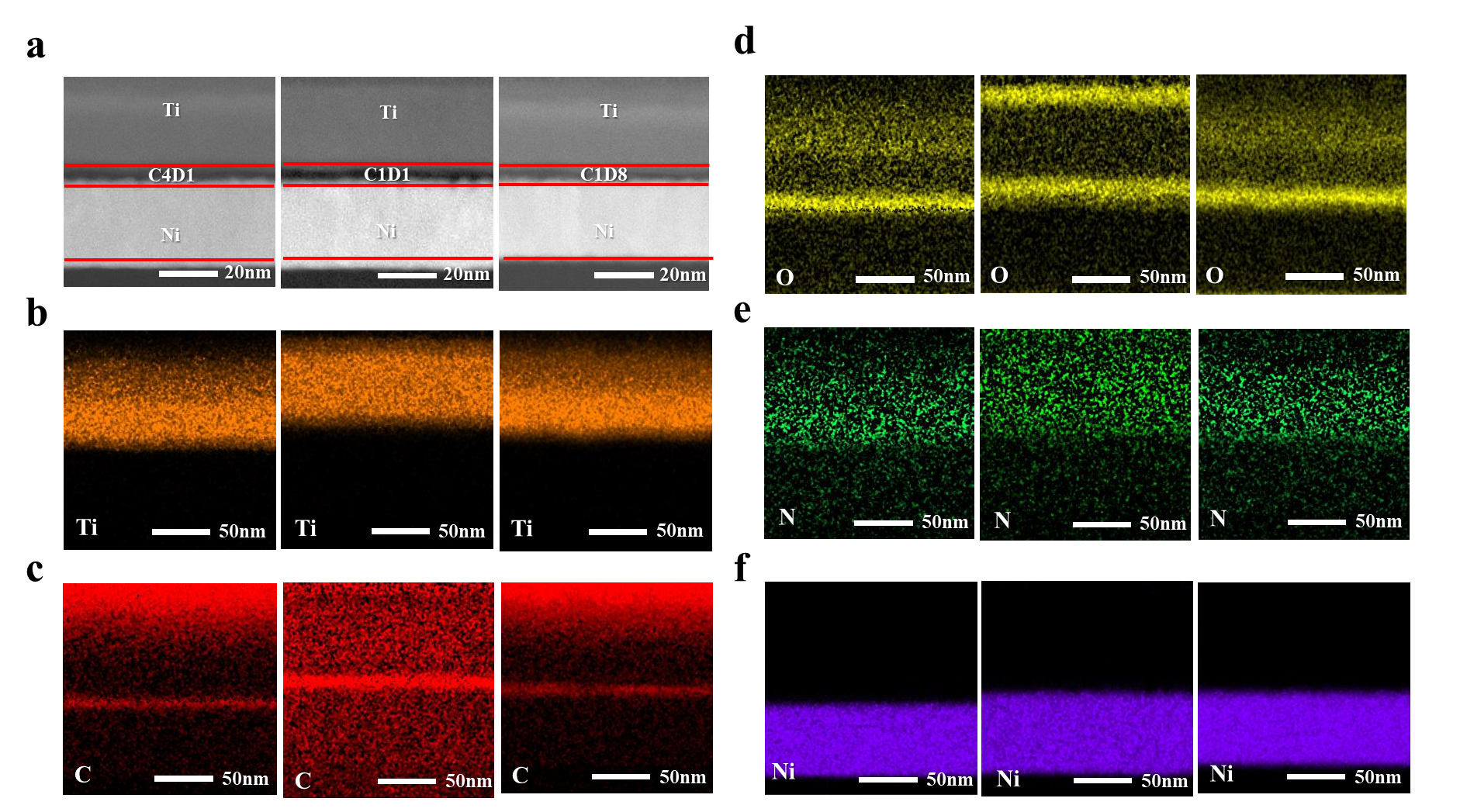


Figure S3. Cross-sectional TEM and EDS mapping images of Ti/p(CEA-co-DEGDVE)/Ni devices (Left: pC4D1, Middle: pC1D1, Right: pC1D8). (a) TEM image, (b) Ti element (TE), (c) C element, (d) O element, (e) N element, (f) Ni element (BE).

Each layer of the Ti/p(CEA-co-DEGDVE)/Ni device is clearly distinguishable. The p(CEA-co- DEGDVE) was measured using a high-resolution transmission electron microscope (TEM) image, as shown in Figure S3(a). and the distribution of elements (Ti, C, O, N, Ni) obtained by energy-dispersive X-ray spectroscopy (EDS) mapping is shown Figure S3(b-f).


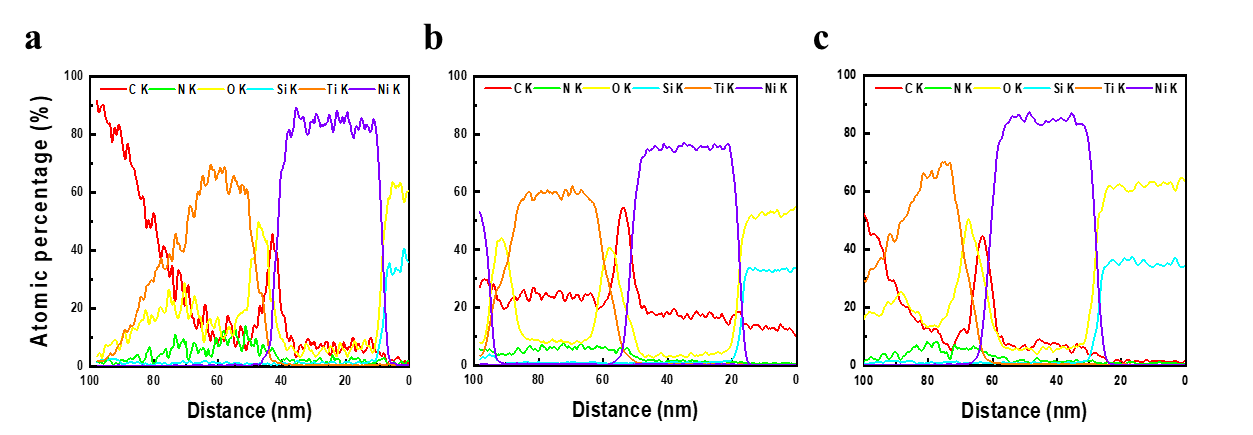


Figure S4. Energy dispersive X-ray spectroscopy (EDS) line scan analysis (a) pC4D1 (b) pC1D1 (c) pC1D8.

To further investigate the elemental distribution and interface uniformity of the fabricated sample, Energy Dispersive X-ray Spectroscopy (EDS) line scan analyses were conducted, as shown in Figure S4(a–c). In Figure S4(a-c), the line scan across the interface demonstrates a well-defined boundary between the adjacent layers, with each element showing a distinct and abrupt transition in its atomic concentration. The atomic concentration profiles remain stable and uniform across the bulk of the film, confirming that the target composition was achieved throughout the depth of the sample. No significant deviations or unexpected elemental inclusions were observed, indicating high compositional precision and process control during synthesis.


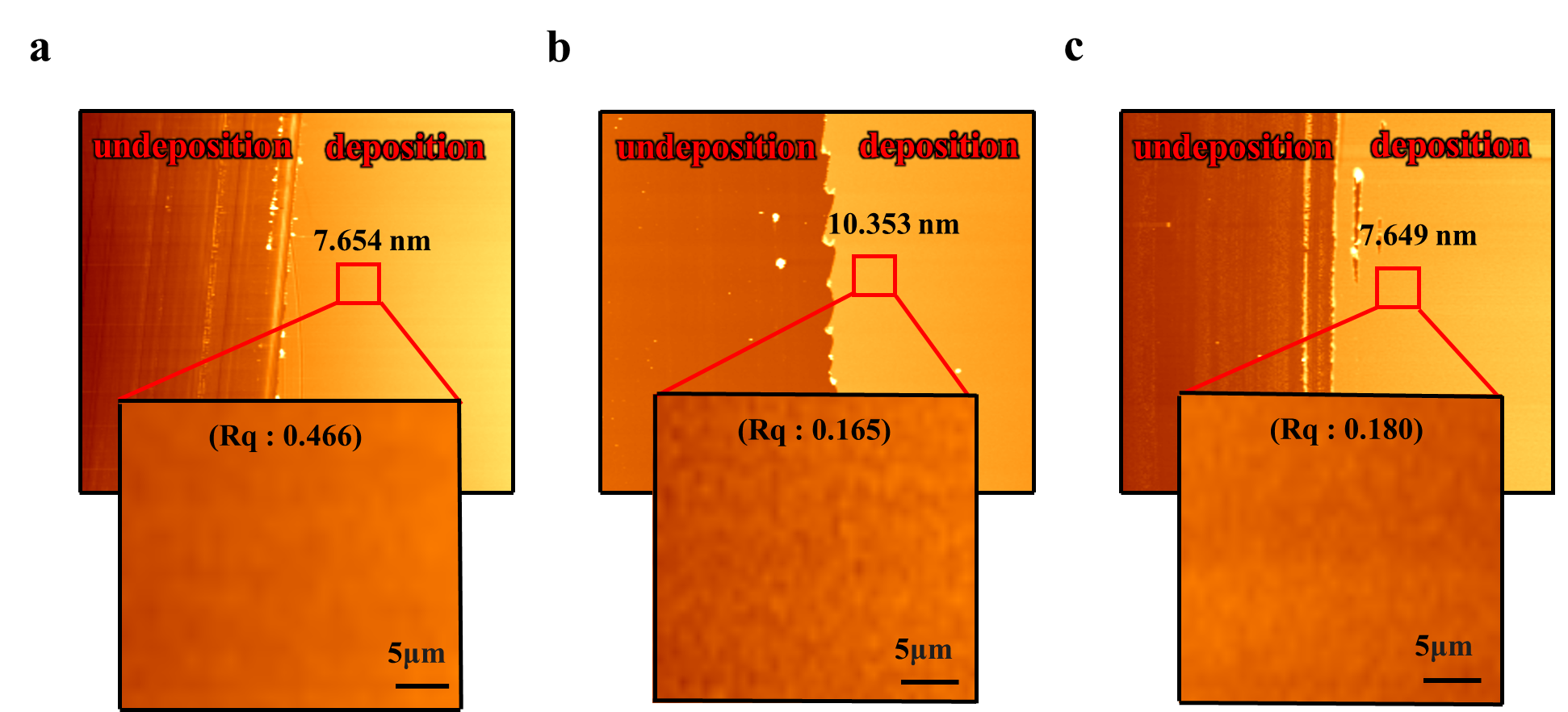


Figure S5. AFM image of a 10 nm thick copolymer prepared by chemical vapor deposition (iCVD) process (a) pC4D1 (b) pC1D1 (c) pC1D8.

Figure S5 shows an AFM image of a 10 nm thick copolymer via a chemical vapor deposition process (iCVD). All three films produced uniform, defect-free copolymer thin films at the nanometer scale. This may be due to the conformal coverage characteristics of not only polymer but also co-polymer films. No phase separation between CEA and DEGDVE was observed, indicating that a homogeneous co-polymer surface was formed. All three films showed excellent root mean square roughness (Rq) of less than 0.5 nm, measured at the indicated locations. Maintaining a smooth surface morphology is an important task to minimize electrical property problems of memristor that may occur on the polymer film surface and to optimize the device.


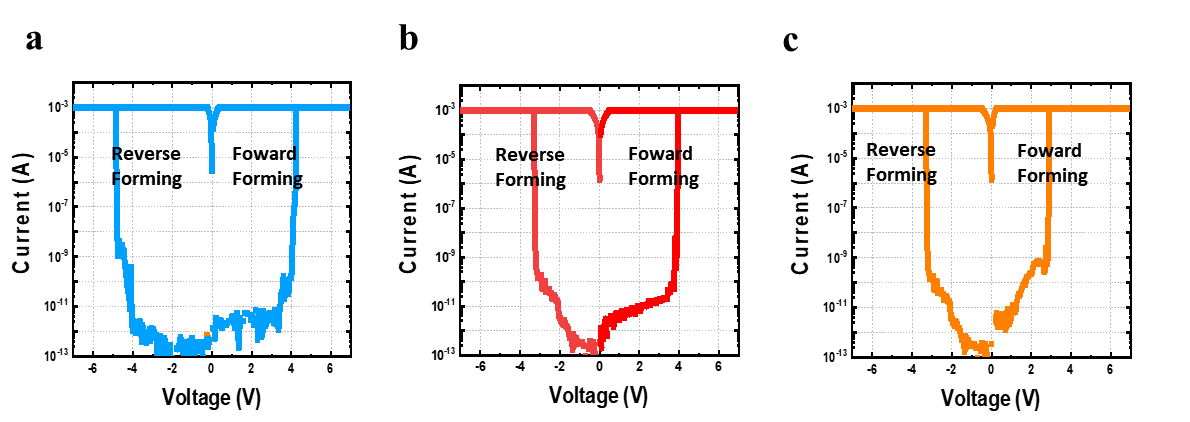


Figure S6. Forming behavior according to (a) pC4D1, (b), pC1D1, and (c) pC1D8.


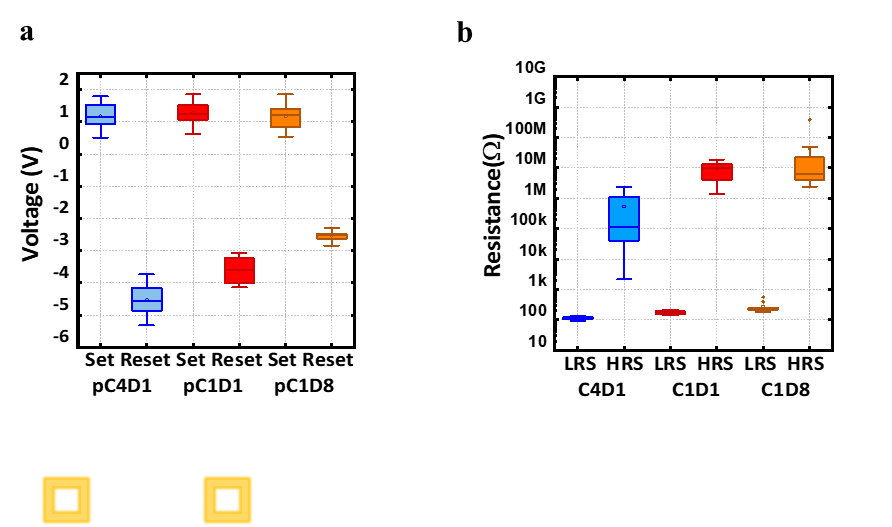


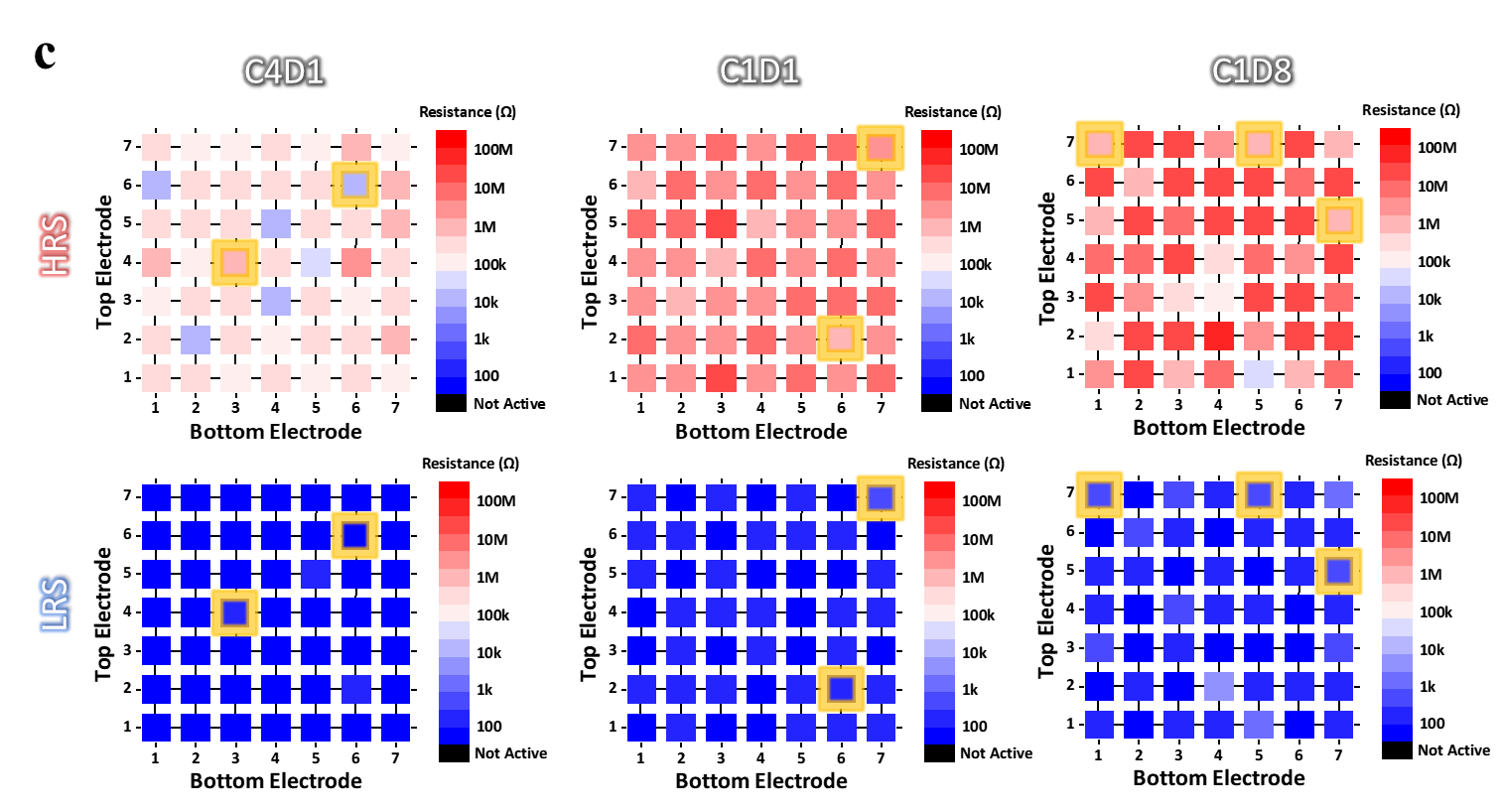


Figure S7. (a) Set, reset box plot according to operating voltage composition ratio (b) Box plot distribution of LRS and HRS resistance (c) Spatial distribution maps of HRS and LRS states in cell arrays under different material compositions.


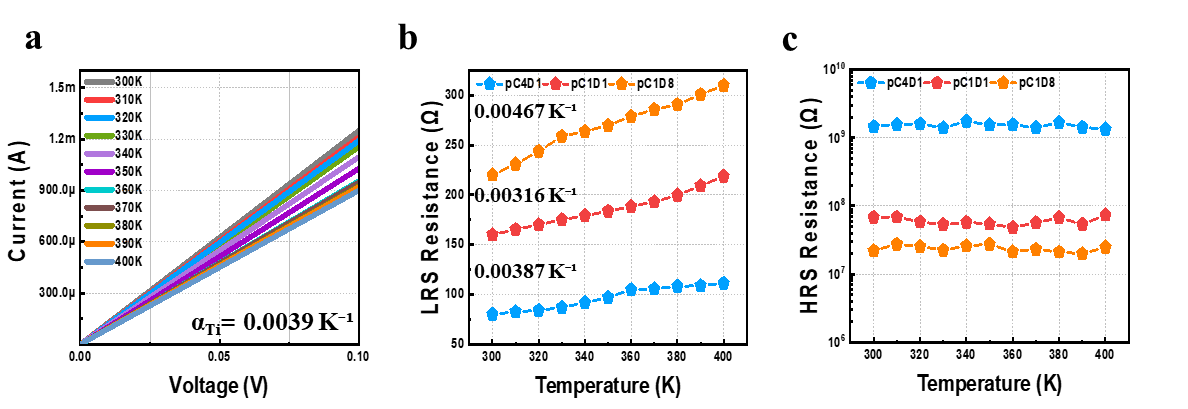


Figure S8. (a) I-V plot of the LRS (b) Changes in LRS temperature coefficient according to composition ratio (c) Changes in HRS temperature coefficient according to composition ratio.

First, the typical linear temperature dependence of the conducting filaments in metallic behavior,

$$R\left( T \right)=R_{0}[1+\alpha\left( T-T_{0} \right)]$$

Figure S8a shows that the p(CEA-co-DEGDVE) film-based device shows a current decrease with increasing temperature. The LRS resistance of the device was considered from 300 to 400 K. The calculated temperature coefficient of resistance (γ) is about 0.00387 K⁻¹. This is similar to the temperature coefficient of titanium of 0.0039 K⁻¹, which confirms that the formation and destruction of conductive filaments are affected by titanium. Figure S8b shows the LRS resistance change according to the composition ratio. Although there are some differences depending on the composition, they all show a consistent temperature dependence of titanium. In addition, the HRS resistance change according to temperature shows the characteristic of temperature independence. Figure S8c presents a schematic illustration suggesting that the upper electrode titanium is the main cause of filament behavior. Based on the temperature-dependent resistance behavior and schematic illustration results, we propose that the top electrode titanium is the major contributing factor to the filamentary conduction path, supporting the hypothesis that titanium intrusion is responsible for the formation of metallic filaments.


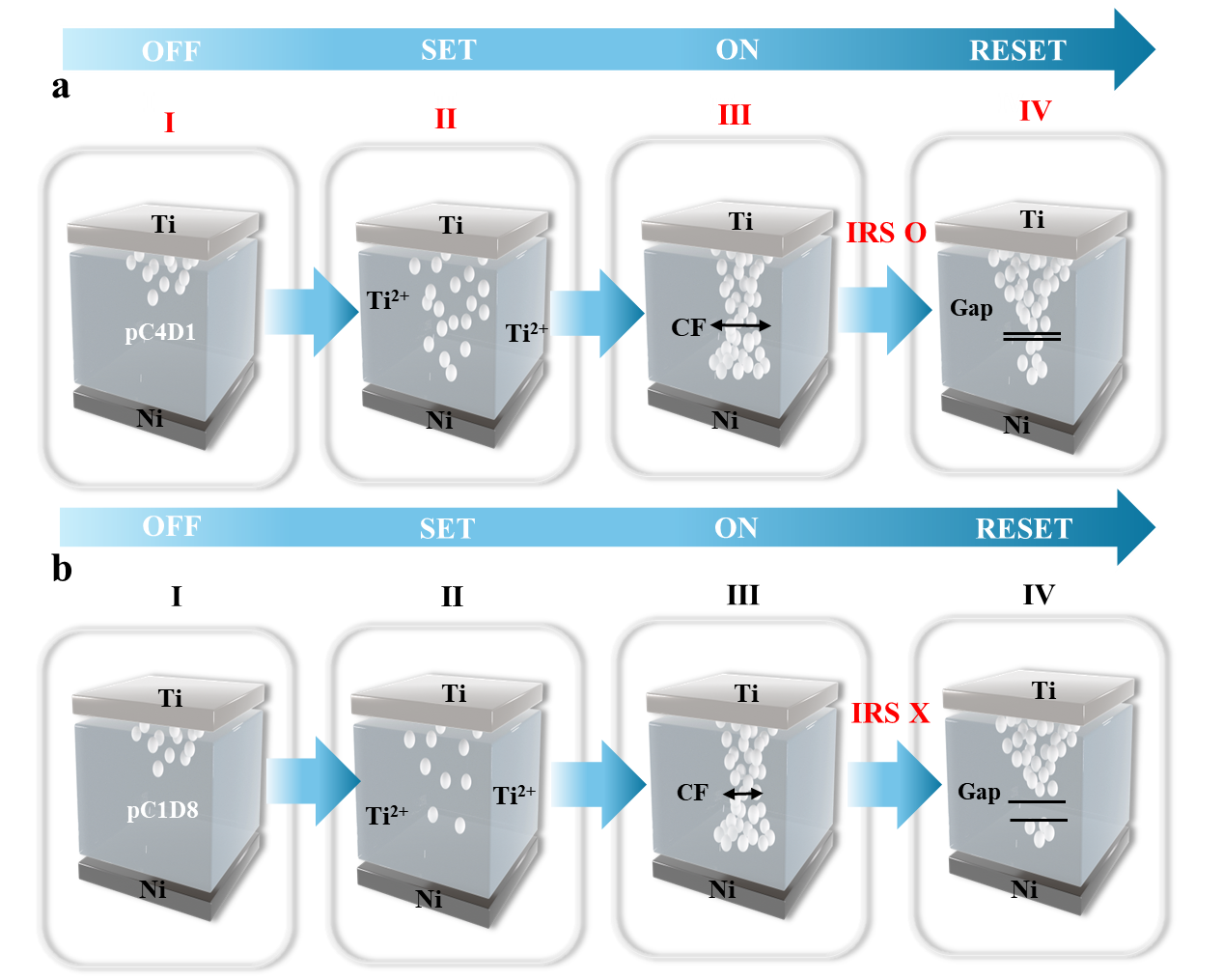


Figure S9. (a) Schematic illustration of filament formation and destruction (thick filaments) with increasing CEA composition ratio. (b) Schematic illustration of filament formation and destruction (thin filaments) with increasing DEGDVE composition ratio.

As shown in Figure S9a, when the CEA concentration is high and the DEGDVE concentration is low, the forward bias applied to the Ti top electrode (TE) induces the oxidation of Ti atoms, leading to the injection of Ti cations (Ti^2^⁺) into the resistive switching layer (Figure S9a, step 1). Due to the strong polarity of the cyano (-CN) functional groups, the interaction between the metal cations and the polymer matrix significantly limits the ion mobility. This decrease in mobility causes the metal cations to accumulate at specific sites, resulting in a highly localized switching region with an increased metal ion concentration (Figure S9a, step 2). As a result, thick conductive filaments (CF) are formed at these sites (Figure S9a, step 3). Conversely, when the CEA concentration is low and the DEGDVE concentration is high (Figure S9b, the high cross-linking density due to DEGDVE limits the metal ion implantation (Figure S9b, step 1). In addition, the weaker metal-polymer interaction leads to higher ion mobility, which leads to a more uniform distribution of metal ions and less localized switching sites (Figure S9b, step 2). As a result, this process facilitates the formation of thinner and weaker conducting filaments (Figure S9b, step 3). During the RESET process, these filaments are prone to complete rupture, effectively eliminating the residual conducting path and ensuring a stable OFF state (Figure S9b, step 4). For high CEA, when a negative bias is applied, the thick CF formed in step 3 is not completely ruptured during the RESET process (Figure S9a, step 4).


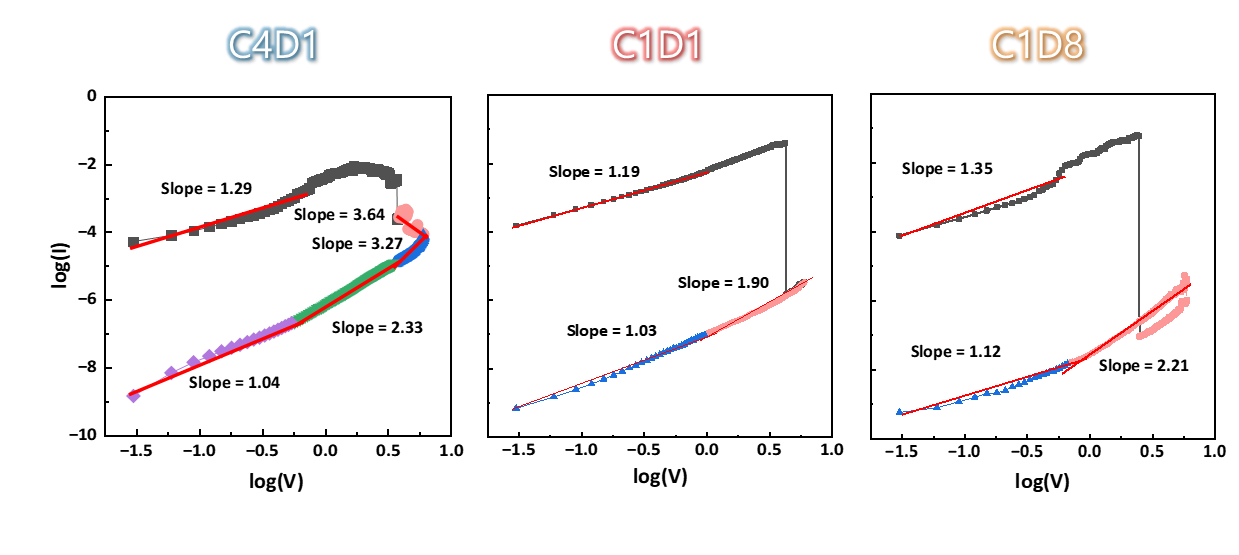
 Figure S10. SCLC-based conduction analysis of memristor devices with varying material compositions. (left) C4D1, (middle) C1D1, (right) C1D8.

As shown in Figure S10, the I–V curves for different compositions were replotted on a double logarithmic scale. In the early stage of the RESET process, an ohmic conduction regime is observed, indicating that the conductive filament is still intact. This is followed by regions with slopes greater than 2, which can be attributed to trap-controlled space-charge-limited conduction (SCLC) as the filament ruptures, where the steep current-increasing region at higher voltage indicates that detrapped carriers together with newly injected free carriers contribute to conduction, leading to relatively higher HRS current levels in pC4D1. Notably, the higher cyano content in pC4D1, as qualitatively confirmed by XPS atomic concentration and FTIR intensity analyses (Figures 2b,d) is consistent with its enhanced trap-related conduction behavior.[1,2] The highly polar cyano functional groups in the CEA monomer provide additional trap sites within the switching layer, which can contribute to non-ideal behaviors such as intermediate resistance states (IRS). The presence of such high-slope SCLC regions in pC4D1 supports the interpretation that enhanced trapping effects are responsible for its higher tendency to exhibit IRS behavior compared to other compositions. This trap-assisted conduction is also reflected in the potentiation–depression curves (Figure S14a), where pC4D1 tends to show greater nonlinearity and asymmetry, leading to less ideal synaptic weight updates.


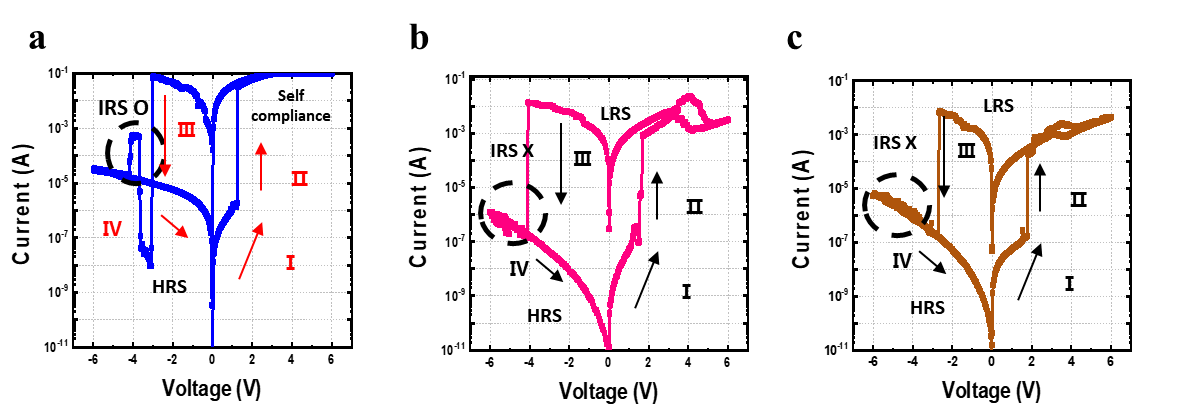


Figure S11. Bipolar behavior based on Al/p(CEA-co-DEGDVE)/Ni (a) pC4D1 (b) pC1D1 (c) pC1D8.


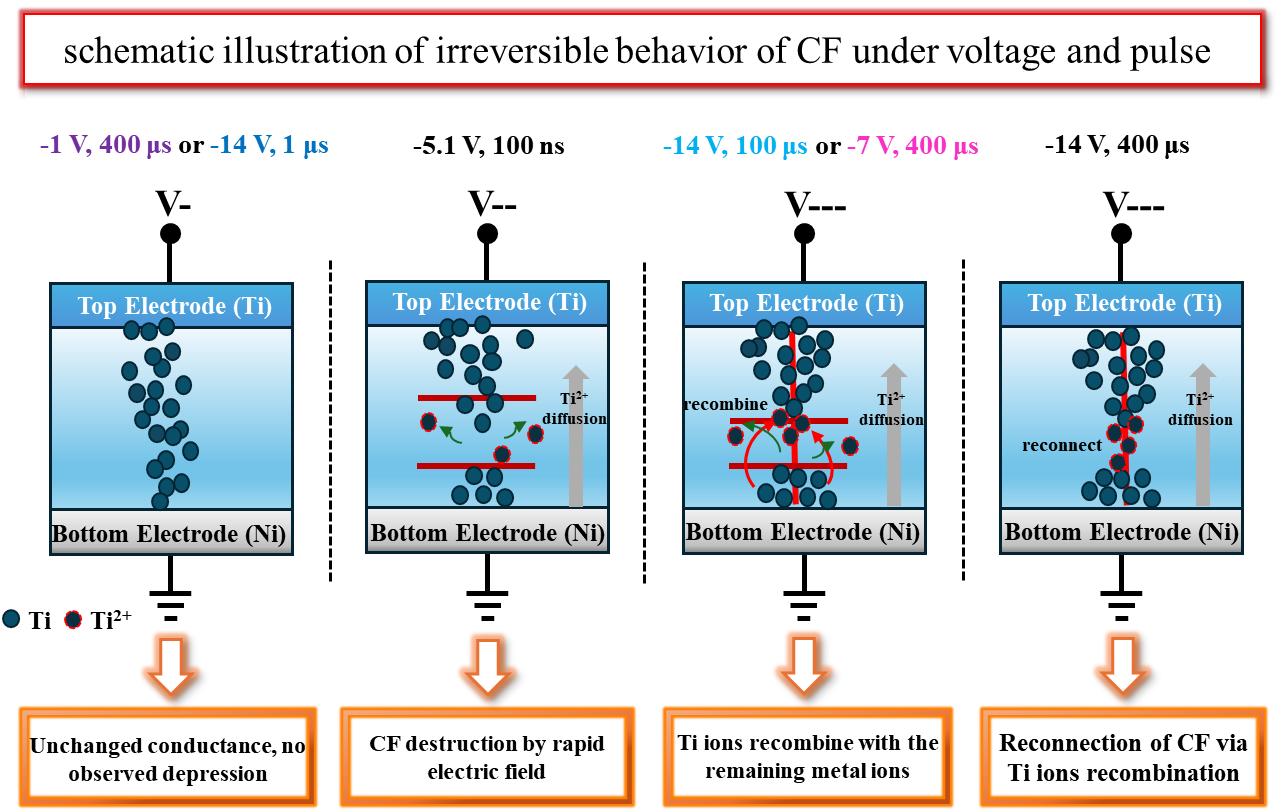


Figure S12. Schematic illustration of filament modulation according to electric field.


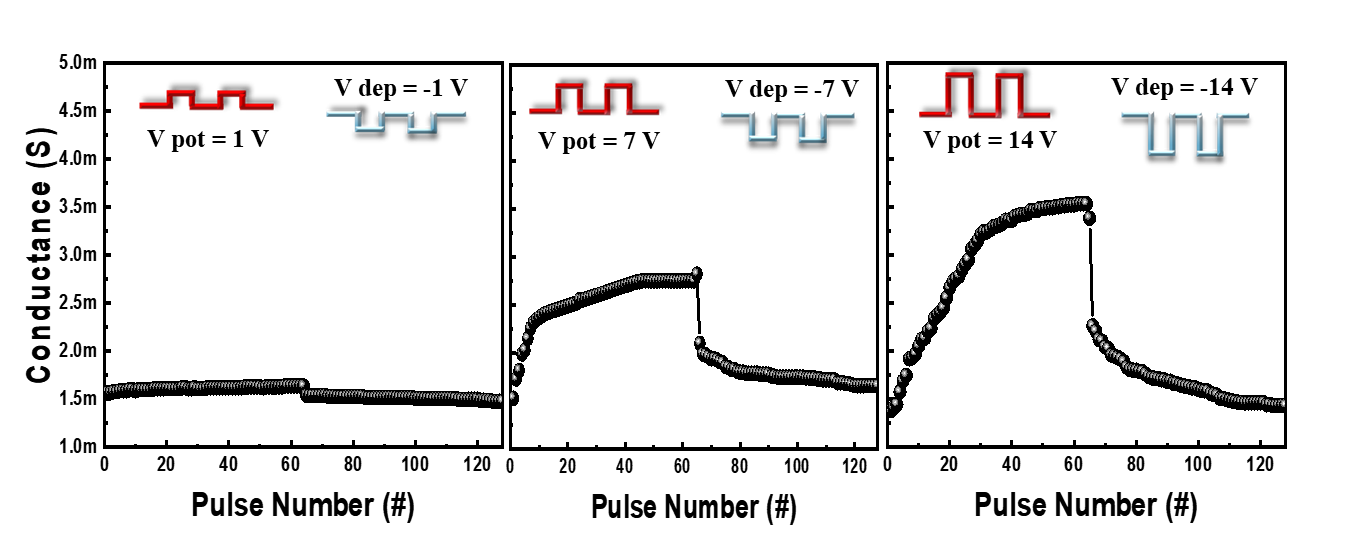
 Figure S13. Conductance modulation of the p(CEA-co-DEGDVE) memristor under different applied voltages (left): 1 V/－1 V, (middle): 7 V/－7 V, (right): 14 V/－14 V.


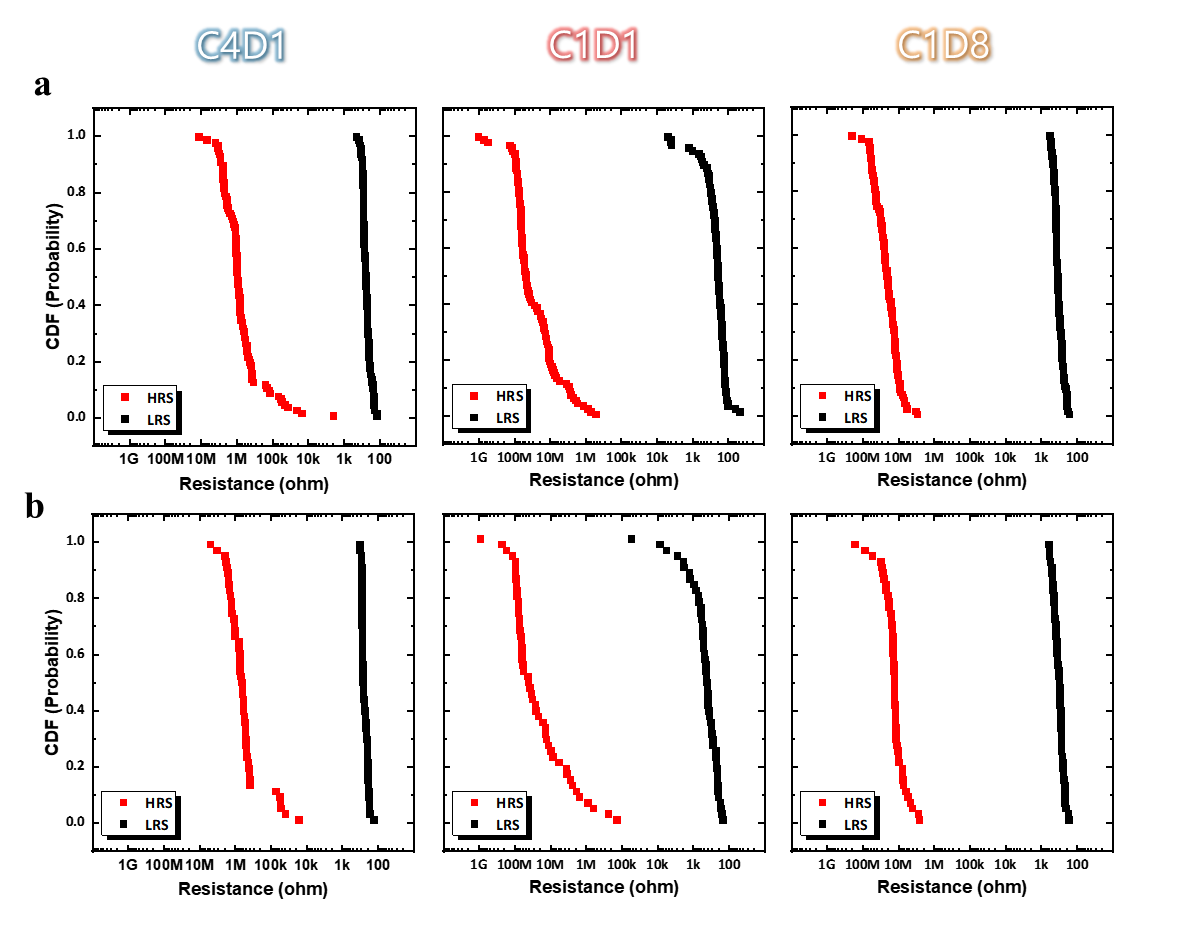


Figure S14. Cumulative distribution functions (CDF) of LRS and HRS resistance over 100 switching cycles measured at a read voltage of 0.1 V for (a) cycle-to-cycle (C-to-C) variability and (b) device-to-device (D-to-D) variability collected from 100 cycles of a fabricated 7 × 7 array in pC4D1, pC1D1, and pC1D8 memristor devices.

Table S2. Statistical parameters of high-resistance state (HRS) and low-resistance state (LRS) for pC4D1, pC1D1, and pC1D8 devices obtained from cycle-to-cycle (C-to-C) variability analysis over 100 switching cycles.

| parameter | C4D1 | | C1D1 | | C1D8 | |
| --- | --- | --- | --- | --- | --- | --- |
|  | HRS | LRS | HRS | LRS | HRS | LRS |
| $\sigma$ | 1.29M | 240.79 | 129.32 | 800.77 | 28.17 | 339.48 |
| $\mu$ | 1.44M | 59.26 | 64.38M | 386.52 | 25.49M | 92.93 |
| $CV$ | 1.117 | 0.246 | 2.007 | 2.072 | 0.905 | 0.274 |
| $M_{i\_worst}$[dB] | 12.16 | | 37.36 | | 42.62 | |

Table S3. Statistical parameters of HRS and LRS for pC4D1, pC1D1, and pC1D8 devices obtained from device-to-device (D-to-D) variability analysis in a 7x7 devices array of over 100 switching cycles.

| parameter | C4D1 | | C1D1 | | C1D8 | |
| --- | --- | --- | --- | --- | --- | --- |
|  | HRS | LRS | HRS | LRS | HRS | LRS |
| $\sigma$ | 0.89M | 238.32 | 66.03M | 1.86k | 18.58M | 339.36 |
| $\mu$ | 0.82M | 44.08 | 128.83M | 7.54k | 24.31M | 111.04 |
| $CV$ | 0.923 | 0.185 | 1.951 | 4.045 | 1.309 | 0.327 |
| $M_{i\_worst}$[dB] | 20.86 | | 29.68 | | 41.86 | |


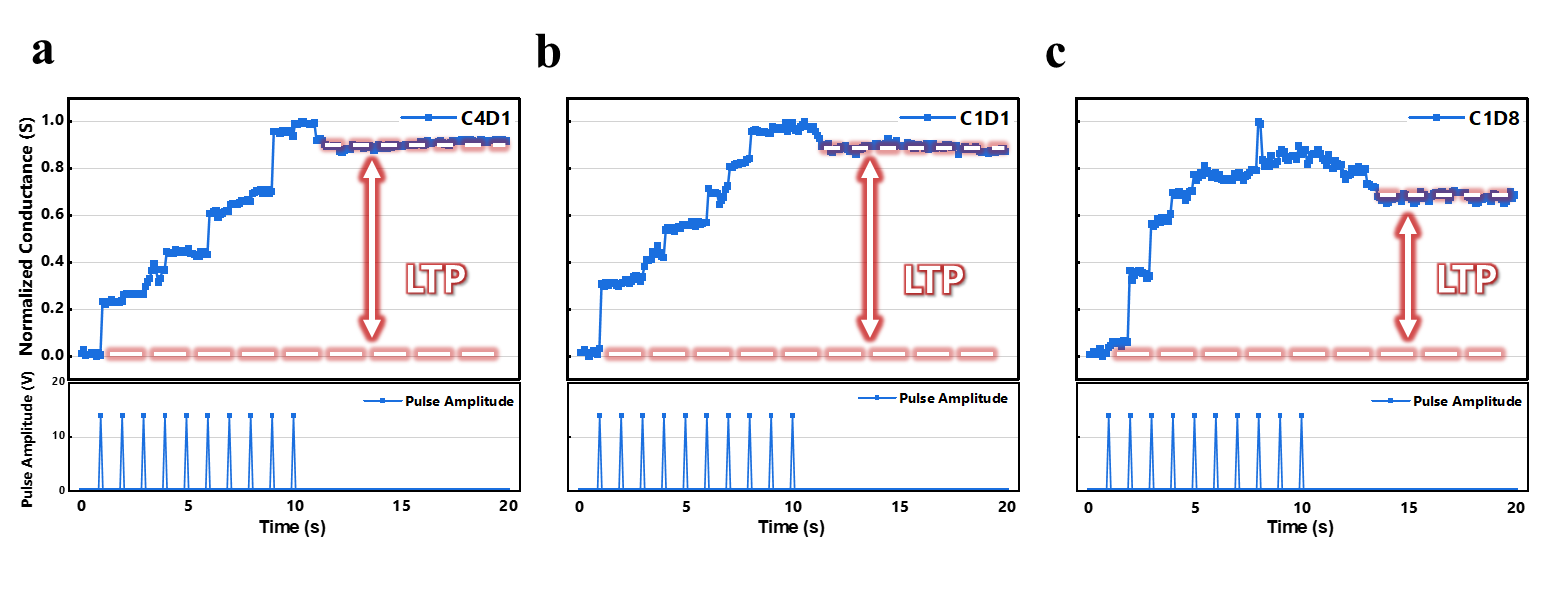


Figure S15. Long-term potentiation (LTP) characteristics of p(CEA-co-DEGDVE) memristors measured under 14 V, 400 μs pulse trains (read at 0.1 V) with different compositions.

**
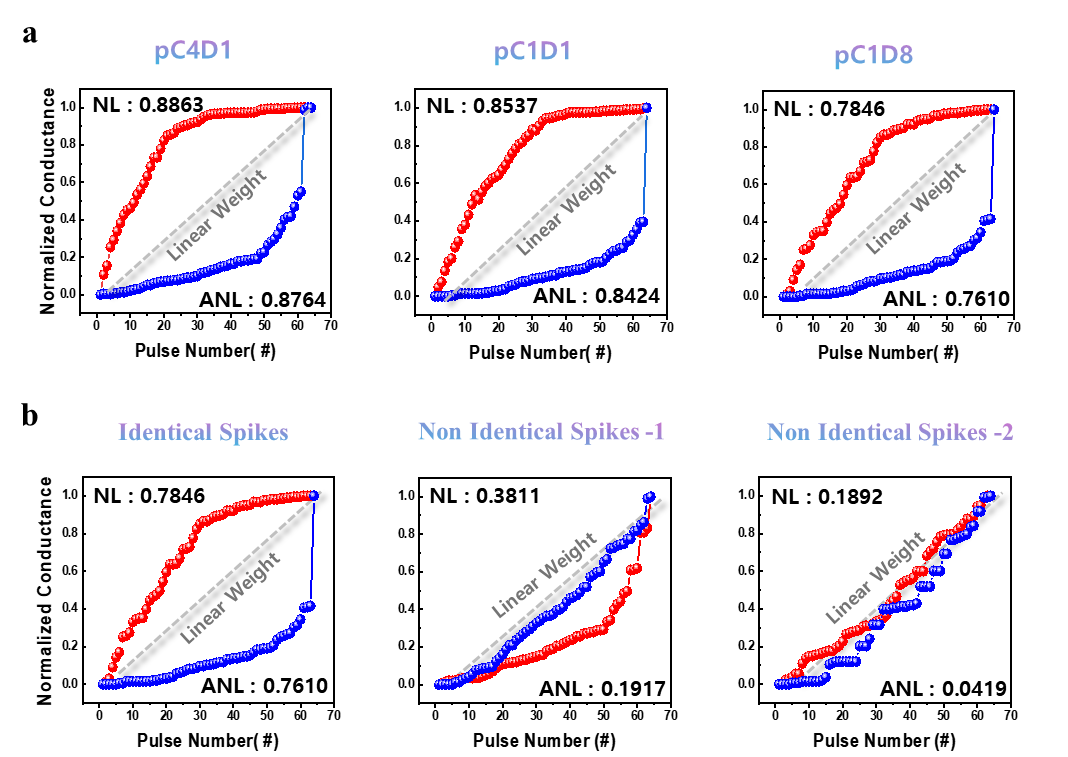
**

Figure S16. (a) Comparison of normalized potentiation-depression curves by composition ratio (b) Comparison of normalized curves by spike shape.


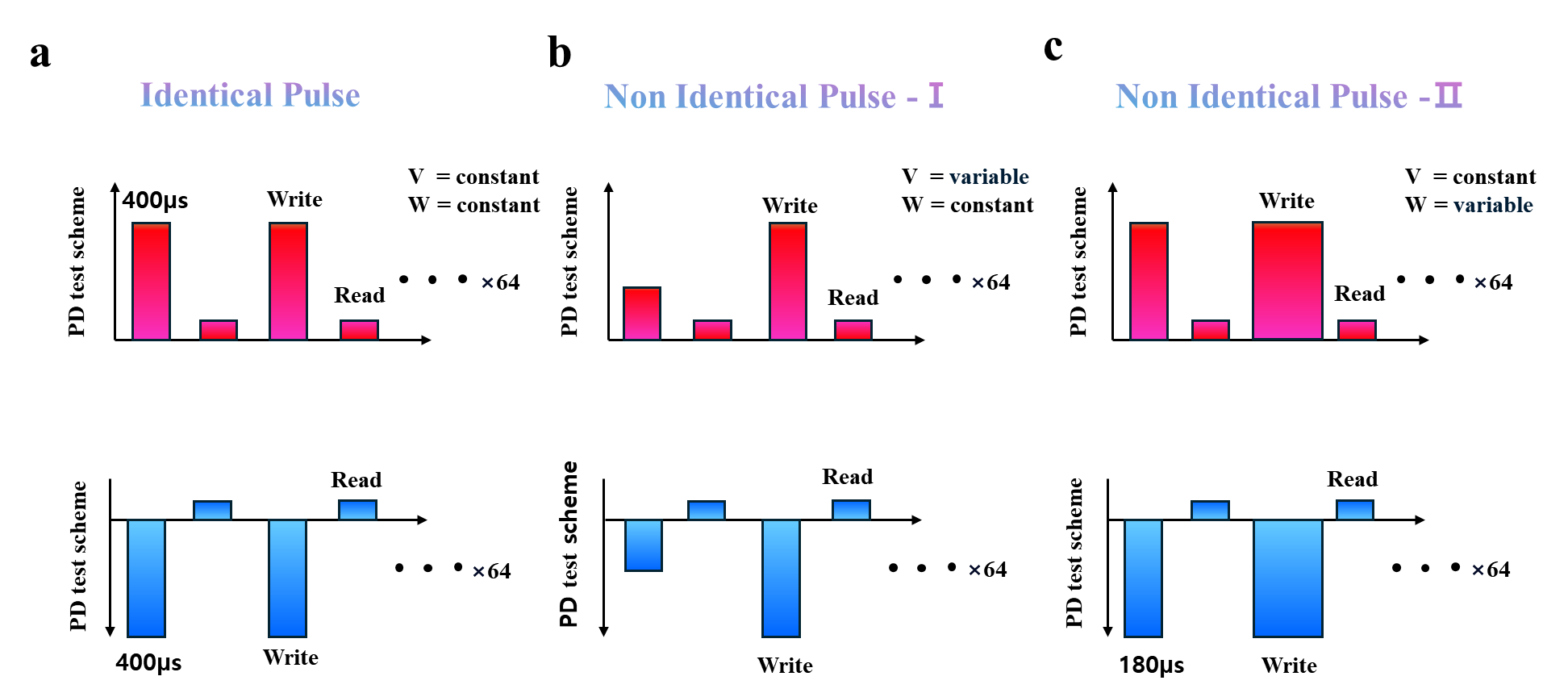
 Figure S17. Comparison of (a) ideal pulse, (b) non-ideal pulse-Ⅰ, and (c) non-ideal pulse-Ⅱ.

Table S4. Comparison of nonlinearities by composition ratio and spike shape

|  | Identical spikes | Non identical spikes-Ⅰ | Non identical spikes-Ⅱ |
| --- | --- | --- | --- |
| Pulse Amplitude (V) | Potentiation : 14  Depression: -14 | Potentiation : 6~14  Depression: -6 to -14 | Potentiation : 14  Depression: -14 |
| Pulse period (μs) | 400.1 | 400.1 | 400.1 |
| Pulse width(μs) | 400 | 400 | 180~500 |
| Step | X | 0.125V | 5μs |
| Read Voltage (V) | 0.1 | 0.1 | 0.1 |


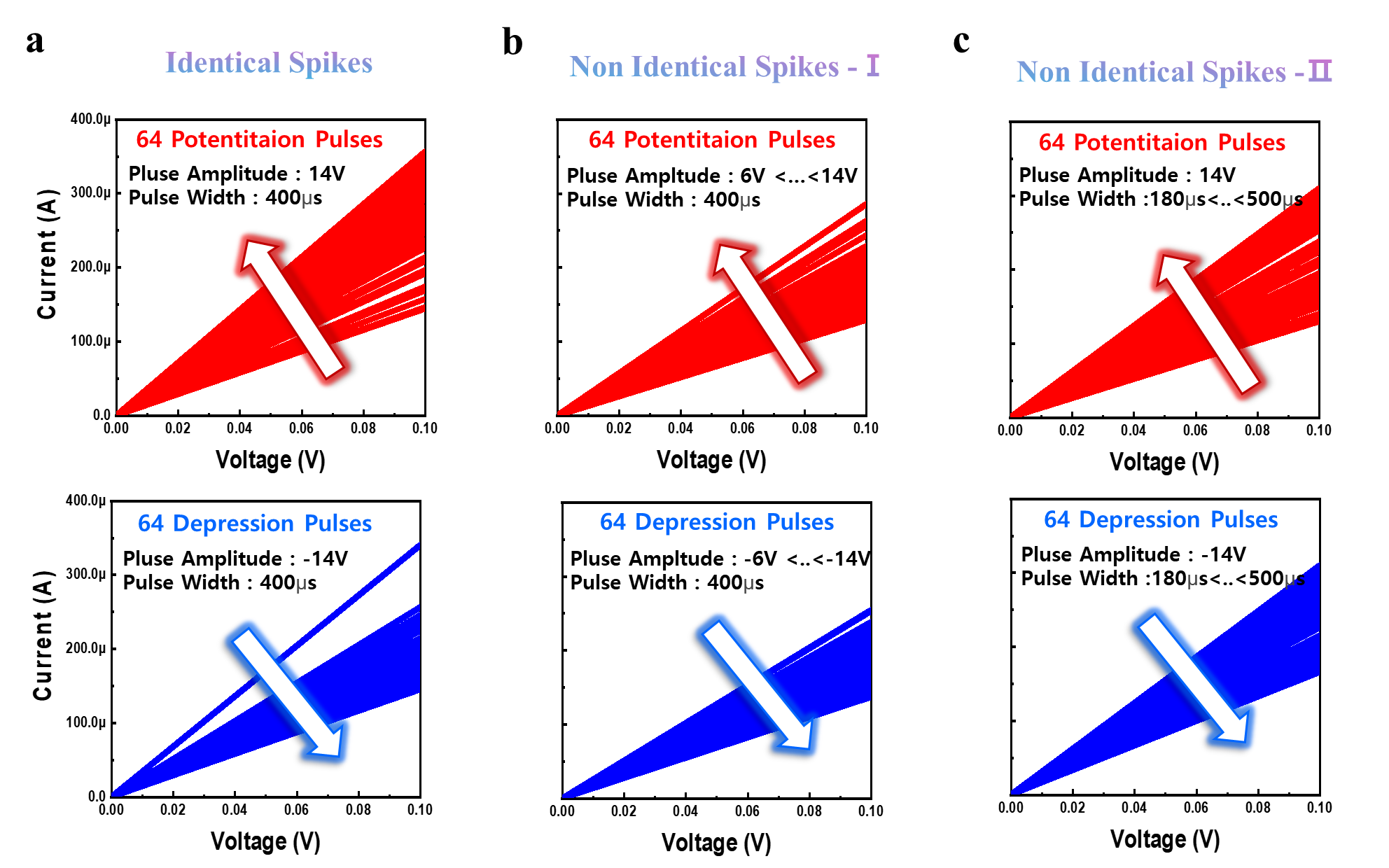


Figure S18. Differences in I-V curves of potentiation and depression according to pulse spike shape (a) identical spikes (b) non-identical spikes-Ⅰ (c) non-identical spikes-Ⅱ.

The performance of memristor devices is highly dependent on the shape of the pulse signal, especially the amplitude, width, and period, and analyzing the effects of these pulse characteristics on the I-V characteristics of the device is essential for efficient operation of the device. In this study, we experimentally analyzed the changes in the I-V characteristics of the device's potentiation and depression according to various pulse shapes.

Table S5. Comparison of model accuracy by structure and nonlinearity

| Device structure | Neural network model | NL value | ANL value | Accuracy (%) | Ref. |
| --- | --- | --- | --- | --- | --- |
| Ti/p(CEA-co -DEGDVE)/Ni | CNN | 0.189 | 0.042 | 88.1 | This work |
| Al/Ga2O3:Sn/Sapphire | N/A | 0.35 | 0.0290 | 97.3 | ^[3]^ |
| Ag/Pd/SiGe/Si | N/A | 0.5 | 0.0219 | 95.1 | ^[4]^ |
| Au/LixMoS2/Au | N/A | 0.3 | 0.188 | N/A | ^[5]^ |
| Ta/TaOx/TiO2/Ti | HNN | 0.6-0.81 | 0.52-0.82 | 70~80 | ^[6]^ |
| Au/P(VDF-TrFE)/NSTO | N/A | 0.6 | 0.866 | N/A | ^[7]^ |
| Cu/pV3D3/Al | SNN | 0.71 | 0.625 | 86 | ^[8]^ |
| Cu/p(V3D3-co-VI)/Al | DNN | 0.726 | 0.056 | 94 | ^[9]^ |
| ITO/PEDOT:PSS/Al | SNN | 0.574 | 0.563 | 95.2 | ^[10]^ |


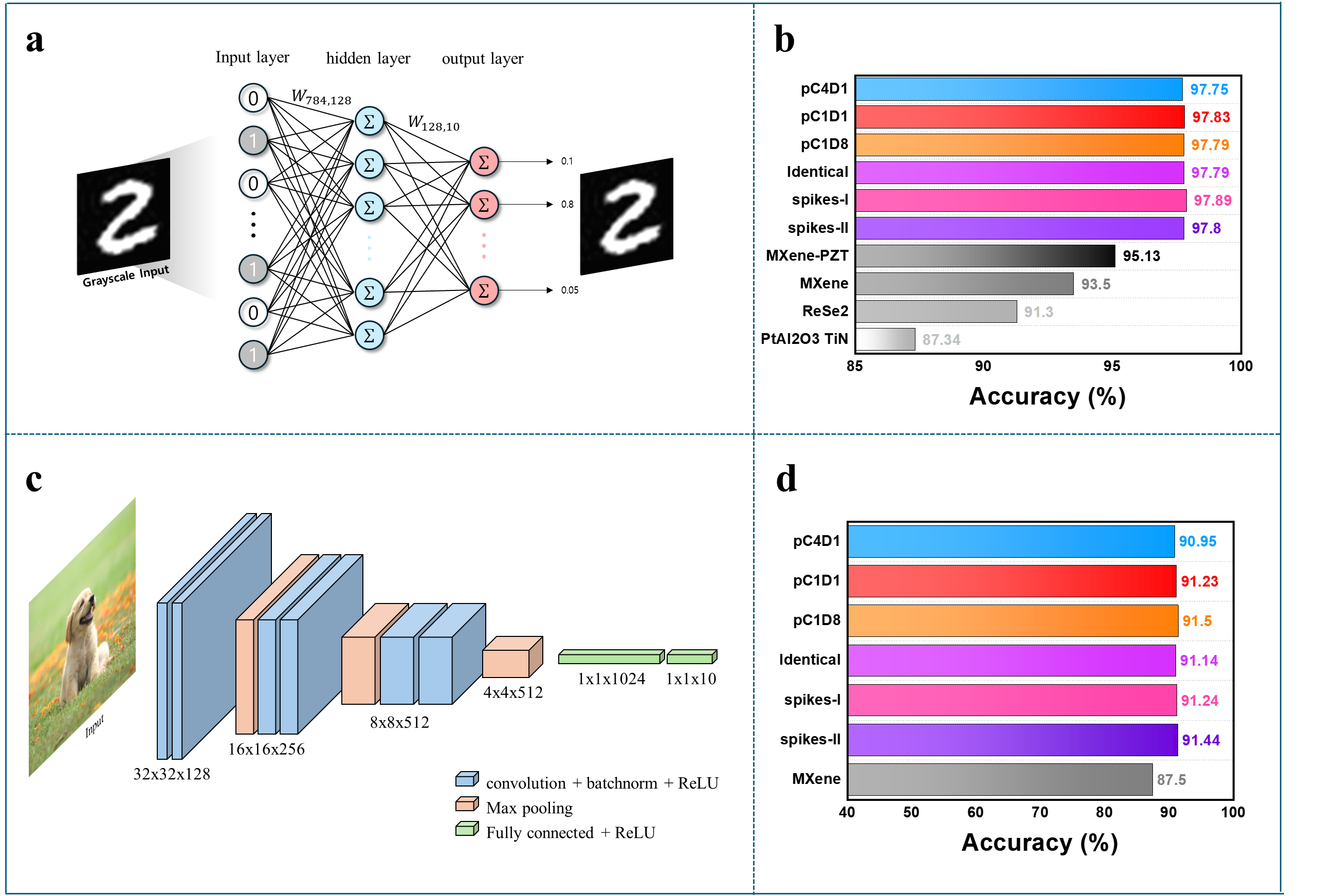


Figure S19. (a) MLP architecture for MNIST classification (b) Results of experiment on MNIST dataset (c) CNN architecture for CIFAR-10 classification (d) Results of experiment on CIFAR-10 dataset.

Our approach consistently delivered high classification accuracy across both low- and high-resolution benchmarks. On MNIST, the model achieved over 97% accuracy (Figure S19b), comparable to state-of-the-art software-based implementations, confirming that the proposed framework preserves accuracy even under device-aware constraints. On CIFAR-10, it attained more than 90% accuracy (Figure S19d), representing a clear improvement over conventional baselines and demonstrating robustness on more complex natural images.

All experiments were conducted using the PyTorch framework on two NVIDIA RTX 4090 GPUs. Each model was trained for 200 epochs with a batch size of 256, and every experiment was repeated three times with different random seeds. We report the mean accuracy across runs to reduce the influence of randomness.

For the MNIST and CIFAR-10 datasets, we followed standard practice to ensure fair comparison with prior work.^[11-14]^ Specifically, on MNIST, we used a multilayer perceptron (MLP) consisting of an input layer with 784 neurons, a hidden layer with 128 neurons, and a 10-class output layer (Figure S19a). On CIFAR-10, we adopted a conventional convolutional neural network (CNN) architecture comprising sequential convolution, pooling, and fully connected layers (Figure S19c).

For the high-resolution datasets (Oxford 102 Flowers, Stanford Cars, and Food-101), we employed architecture consistent with ResNet-style training protocols. During training, images were randomly resized and cropped to 224×224 pixels, normalized by channel mean and variance, and augmented using random horizontal flips and color jitter. These transformations follow the standard data augmentation scheme described in the ResNet paper. Convolutional architectures can process variable-sized inputs, and a global average pooling layer ensures that the final feature representation matches the classification layer. This approach preserves high-resolution details while maintaining compatibility with the trained network.

Unless otherwise noted, we report top 1 classification accuracy on the official test split (or the defined split for Oxford 102 Flowers) after 200 training epochs.


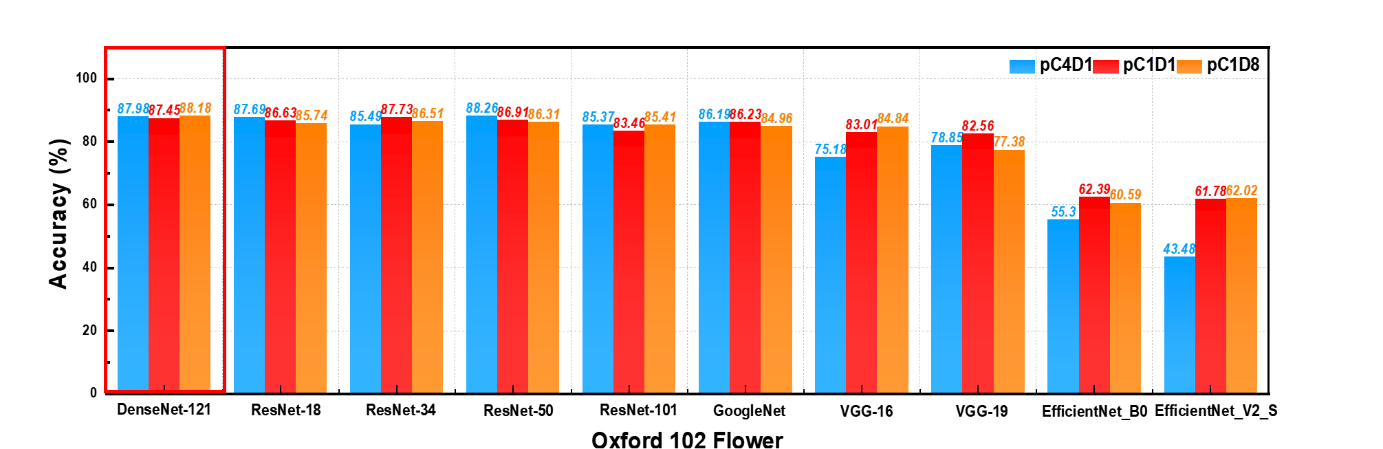


Figure S20. Baseline model performance comparison on Oxford 102 Flower.

We employed a range of widely used deep neural networks, including VGG16, VGG19, GoogleNet, ResNet-X, DenseNet-121, EfficientNet-B0 and EfficientNetV2-S.^[15-20]^ As shown in Figure S20, the proposed device achieved high classification accuracy across all tested architectures, demonstrating its compatibility with a variety of deep learning models.

Based on these results, DenseNet-121, which achieved the highest accuracy, was selected as the baseline model for subsequent evaluations on other benchmark datasets (i.e., Stanford Cars ^[21]^ and Food-101^[22]^)

Table S6. Classification accuracy (%) comparison to analyze the contribution of linear and non-linear components.

| Model | Dataset | Linear | Non-linear | Linear +  Non-linear |
| --- | --- | --- | --- | --- |
| DenseNet-121 | Oxford 102 Flower | 87.08 | 86.68 | **87.98** |
|  | Stanford Cars | 74.97 | 71.85 | **75.33** |
|  | Food-101 | 77.57 | 76.79 | **79.13** |
| ResNet-101 | Oxford 102 Flower | 84.68 | 84.80 | **85.37** |
|  | Stanford Cars | 73.84 | 70.91 | **75.61** |
|  | Food-101 | 78.91 | 77.64 | **80.16** |

As shown in Figure 4c, the device exhibits improved conductance linearity within the first ~32 pulses. To examine whether restricting operation to this linear region benefits CNN performance, we compared three modes: (i) Linear-only updates, (ii) Non-linear-only updates, and (iii) a combined scheme utilizing both regions. The results in Table S6 show a clear trend. Linear-only operation consistently outperformed Non-linear-only operation across all datasets, confirming the advantage of stable weight updates. More importantly, the combined Linear + Non-linear scheme achieved the highest accuracies across DenseNet-121, ResNet-101, and GoogleNet. This indicates that, while linear updates ensure stability, the additional variability introduced by non-linear conductance modulation enriches the representational capacity of the network, ultimately leading to superior overall performance. These findings suggest that restricting the number or duration of pulses to remain within the linear region is suboptimal. Instead, leveraging both linear and non-linear regimes provides a more effective balance between stability and expressiveness, thereby maximizing classification accuracy.

References

[1] D. K. Tran, S. M. West, A. E. Stewart, W. Kaminsky, S. A. Jenekhe, " Mixed Ionic‐Electronic Transport in A Series of New Cyano‐Functionalized N‐Type Conjugated Ladder Copolymers," *Advanced Functional Materials* **2025**, e10945.

[2] J. Choi, J. Yoon, M. J. Kim, et al., "Spontaneous Generation of a Molecular Thin Hydrophobic Skin Layer on a Sub-20 nm, High-k Polymer Dielectric for Extremely,"*ACS Applied Materials & Interfaces* **2019**, *11* (32), 29113.

[3] P. Li, X. Shan, Y. Lin, et al.,"Tin Doping Induced High‐Performance Solution‐Processed Ga2O3 Photosensor toward Neuromorphic Visual System," *Advanced Functional Materials* **2023**, *33* (46), 2303584.

[4] S. Choi, S. H. Tan, Z. Li, et al.,"SiGe epitaxial memory for neuromorphic computing with reproducible high performance based on engineered dislocations," *Nature materials* **2018**, *17* (4), 335.

[5] X. Zhu, D. Li, X. Liang, W. D. Lu, "Ionic modulation and ionic coupling effects in MoS2 devices for neuromorphic computing," *Nature materials* **2019**, *18* (2), 141.

[6] I.-T. Wang, C.-C. Chang, L.-W. Chiu, T. Chou, T.-H. Hou, "3D Ta/TaOx/TiO2/Ti synaptic array and linearity tuning of weight update for hardware neural network applications," *Nanotechnology* **2016**, *27* (36), 365204.

[7] S. Majumdar, H. Tan, Q. H. Qin, S. van Dijken, "Energy‐efficient organic ferroelectric tunnel junction memristors for neuromorphic computing," *Advanced Electronic Materials* **2019**, *5* (3), 1800795.

[8] B. C. Jang, S. Kim, S. Y. Yang, et al., "Polymer analog memristive synapse with atomic-scale conductive filament for flexible neuromorphic computing system," *Nano letters* **2019**, *19* (2), 839.

[9] J. Oh, S. Y. Yang, S. Kim, et al., "Imidazole-based artificial synapses for neuromorphic computing: a cluster-type conductive filament via controllable nanocluster nucleation," *Materials Horizons* **2023**, *10* (6), 2035.

[10] T.-Y. Wang, Z.-Y. He, H. Liu, L. et al., "Flexible electronic synapses for face recognition application with multimodulated conductance states," *ACS Applied Materials & Interfaces* **2018**, *10* (43), 37345.

[11] J. H. Ju, S. Seo, S. Baek, et al., "Two‐Dimensional MXene Synapse for Brain‐Inspired Neuromorphic Computing," *Small* **2021**, *17* (34), 2102595.

[12] J.-W. Jang, S. Park, Y.-H. Jeong, H. Hwang, "ReRAM-based synaptic device for neuromorphic computing," in *2014 IEEE International Symposium on Circuits and Systems (ISCAS)* IEEE, **2014**, 1054-1057.

[13] Y. Huang, Y. Gu, X. Wu, et al., "ReSe2-Based RRAM and Circuit-Level Model for Neuromorphic Computing," *Frontiers in Nanotechnology* **2021**, *3*, 782836.

[14] H. Ryu, S. Kim, "Synaptic Characteristics from Homogeneous Resistive Switching in Pt/Al2O3/TiN Stack," *Nanomaterials* **2020**, *10* (10), 2055.

[15] K. Simonyan, A. Zisserman, "Very deep convolutional networks for large-scale image recognition," *arXiv preprint arXiv:1409.1556* **2014**.

[16] C. Szegedy, W. Liu, Y. Jia, et al., "Going deeper with convolutions," in *Proceedings of the IEEE conference on computer vision and pattern recognition* **2015**, 1-9.

[17] K. He, X. Zhang, S. Ren, J. Sun, "Deep residual learning for image recognition," in *Proceedings of the IEEE conference on computer vision and pattern recognition* **2016**, 770-778.

[18] G. Huang, Z. Liu, L. Van Der Maaten, K. Q. Weinberger, "Densely connected convolutional networks," in *Proceedings of the IEEE conference on computer vision and pattern recognition* **2017**, 4700-4708.

[19] M. Tan, Q. Le, "Efficientnet: Rethinking model scaling for convolutional neural networks," in *International conference on machine learning* PMLR, **2019**, 6105-6114.

[20] M. Tan, Q. Le, "Efficientnetv2: Smaller models and faster training," in *International conference on machine learning* PMLR, **2021**, 10096-10106.

[21] K. Valev, A. Schumann, L. Sommer, J. Beyerer, "A systematic evaluation of recent deep learning architectures for fine-grained vehicle classification," in *Pattern Recognition and Tracking XXIX* SPIE, **2018**, 1064902.

[22] L. Bossard, M. Guillaumin, L. Van Gool, "Food-101–mining discriminative components with random forests," in *European conference on computer vision* Springer, **2014**, 446-461.
